# Comparing Survivors of Cancer in Population-Based Samples With Those in Online Cancer Communities: Cross-sectional Questionnaire Study

**DOI:** 10.2196/19379

**Published:** 2022-03-08

**Authors:** Mies C van Eenbergen, Ruben D Vromans, Lidwine W Tick, Gerard Vreugdenhil, Emiel J Krahmer, Floortje Mols, Lonneke V van de Poll-Franse

**Affiliations:** 1 Department of Research Netherlands Comprehensive Cancer Organisation (IKNL) Utrecht Netherlands; 2 Department of Communication and Cognition Tilburg University Tilburg Netherlands; 3 Department of Medical Oncology Maxima Medical Center Veldhoven Netherlands; 4 Department of Medical and Clinical Psychology Tilburg University Tilburg Netherlands; 5 Division of Psychosocial Research & Epidemiology The Netherlands Cancer Institute Amsterdam Netherlands

**Keywords:** internet use, breast cancer, prostate cancer, lymphoma, gynecological cancer, cancer survivors, online health community

## Abstract

**Background:**

Most Western countries have websites that provide information on cancer and the opportunity to participate in online cancer communities (OCCs). The number of patients with cancer that participate in these OCCs is growing. These patients are relatively easy to approach for research purposes.

**Objective:**

The objective of this study is to determine the differences and similarities between survivors of cancer in population-based samples and survivors participating in OCCs who use the internet in relation to their illness.

**Methods:**

In 2017, we drew a sample of 539 population-based patients and 531 OCC patients. The population-based patients were sent a paper-based questionnaire, and the OCC patients were sent the same questionnaire on the web. In the questionnaire, we asked patients about their sociodemographics, internet use, sources of information, media use, and wishes regarding future internet use for health care–related purposes, and the effect of internet use on their health care consumption.

**Results:**

The response rate of population-based internet users was 47% (233/496), and that of the OCC group was 40.3% (214/531). The OCC group had a significantly higher education level (*P*<.001), was younger (*P*<.001), had more survivors that were employed (*P*<.001), and attached greater importance to the internet (171/214, 79.9% vs 126/233, 54.1%; *P*<.001) and fellow survivors (107/214, 50% vs 60/233, 25.8%; *P*<.001). Compared with the population-based group, the OCC group reported more intensive internet use immediately after diagnosis, during treatment, and during follow-up (*P*<.001 in each case). There were similarities in terms of the relative importance that survivors attach to the various sources of information, the topics on which they seek information, and their wishes for future eHealth possibilities. The OCC group reported a greater need to participate in a web-based class or chat with others (92/214, 43% vs 44/233, 18.9%).

**Conclusions:**

We conclude that survivors who are members of an OCC are not representative of survivors of cancer in general. There are significant differences in sociodemographic characteristics, internet use during their treatment journey, internet search frequency during their cancer journey, and participation wishes. Using web-based information and communication can support shared decision-making and may facilitate the active participation of patients during their treatment. For research purposes, it is important to take the bias in OCC groups into account.

## Introduction

### Background

Over the past decade, an increasing number of people have been using the internet, especially in Western countries such as the Netherlands, where the availability of the internet is very high [1,2]. Many countries have websites that provide not only information on cancer but also the opportunity to participate in an online cancer community (OCC) or be a member of a web-based cancer platform. For example, in the Netherlands, there is Kanker.nl [3]; in the United Kingdom, there is Macmillan [4]; and, in the United States, there is the American Cancer Society (the related community [5]) [6]. On these websites, patients can find information about the various types of cancer and their treatment or treatments, side effects, and long-term effects. Visitors can also create a profile and become members to read, start a blog, or communicate with fellow patients through chat groups and personal messages. Members of such communities or platforms are often asked to be respondents in cancer research [7-9]; however, to the best of our knowledge, there have been no studies that have systematically compared survivors of cancer with a profile in OCCs with those in population-based samples. Are the characteristics, internet use, and wishes of Dutch survivors of cancer who participate in an OCC different from a selection of survivors of cancer from the Netherlands Cancer Registry (NCR)?

Previous studies on internet use among patients with cancer have shown that the number of patients who use the internet for information, communication, and community purposes has increased sharply in recent years [10-12]. However, the topics that interest patients have remained more or less stable over the same period [10]. Differences between patients over time have been found in the extent to which they use the internet. These have been attributed to (1) gender (men use the internet more often than women), (2) age (young people use the internet more than older adults), and (3) education level (highly educated people use the internet more than those with a low level of education) [10,13-17]. Research has shown that women tend to participate in OCCs more often than men [15,18]. The explanation often given is that women are more often caregivers [19], are more active in health issues, and have different needs for emotional support than men [20-22].

Despite patients’ increasingly intensive use of the internet, health care professionals are still their most important source of information [10,23]. In recent years, much has changed in the physician–patient relationship [24]. The former, predominantly paternalistic approach has made way for a more patient-centered approach with attention to shared decision-making and patients’ individual wishes [24]. When a patient with cancer is confronted with late effects and is chronically affected by it, the *patient-as-partner* concept may be most appropriate, whereby the patient is a participating member of the treatment team [24]. To become a partner, a patient must first develop learning, then assessment, and ultimately adaptation practices [25].

The internet may actively contribute to shared decision-making and patient-as-partner practices as patients can use it independently from their health care professionals; it is always available; and it offers every individual option for content, communication, and community involvement. Researchers frequently recruit and look at patients with cancer who participate in OCCs to find out to what extent patients with cancer have these skills. However, to what extent are these patients representative of the entire population of patients with cancer?

### Objective

In this study, we aim to identify the differences and similarities between survivors of cancer who participate in an OCC and population-based samples of survivors of cancer who use the internet in relation to their illness. We believe it is important to know the differences between these 2 groups as many studies are based on data from survivors in the OCC group, which raises the important question of the extent to which these findings generalize to the complete population of survivors of cancer [7-9]. Although this is an important methodological question, it has received very little attention. We hypothesize that there are significant differences between these 2 groups. First, we expect that survivors in the OCC group who use the internet have different sociodemographic characteristics compared with survivors in the population-based group. Second, we expect that survivors in the OCC group use the internet more often and have different wishes for various purposes, including content, communication, community, and eHealth, compared with survivors in the population-based group. Finally, we expect that survivors in the OCC group are more active media users for communication with health care professionals and relatives than survivors in the population-based group.

Many definitions of cancer survivorship have been used. In this paper, we chose to adopt the most frequently used definition that is also applied by the US National Coalition for Cancer Survivorship and Institute of Medicine: “a person is considered to be a cancer survivor from the time of diagnosis through the balance of his or her life” [26].

## Methods

### Ethics Approval

A declaration of no objection was granted by the medical ethics review committee Midden Brabant (NW2016-47).

### Participants

**Figure 1:**
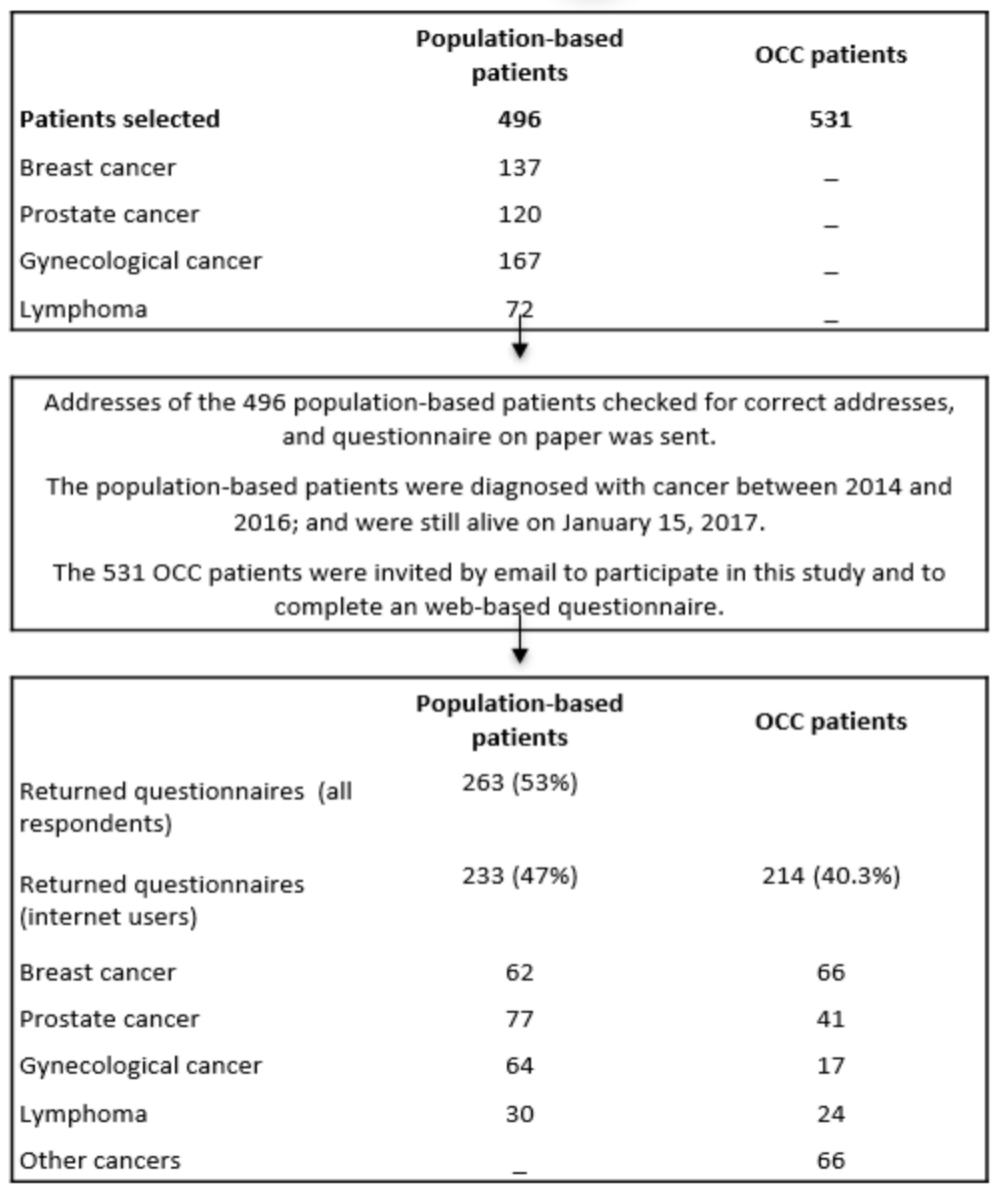
Flowchart of the data collection process. OCC: online cancer community.

For the population-based group, a population-based, cross-sectional survey on internet use was conducted through the NCR. In October 2016, we drew a random sample of 523 patients with breast cancer (138/523, 26.4%), prostate cancer (125/523, 23.9%), gynecological cancer (184/523, 35.2%), or lymphoma (76/523, 14.5%) diagnosed in 4 hospitals in the period between 2014 and 2016 and who were aged ≤70 years at diagnosis. Our samples were linked with the Dutch municipal records database that contains mortality and residential data from all citizens through municipal registries to exclude all deceased patients. Addresses were checked for correctness, and all 496 surviving patients were sent an information letter together with a paper and pencil questionnaire by their oncologist. By replying, the patients explicitly agreed to participate and consented to the linkage of their questionnaire data with their disease history as registered in the NCR. The returned questionnaires were only identifiable by a study number, which guaranteed patient anonymity. We repeated the research method from 2005 to 2017 to describe the changes over time [10]. For the full selection procedure, see Figure 1 and the flowchart in the paper by van Eenbergen et al [10]. For this study, we included only the population-based participants who used the internet.

For the OCC group, in 2017, we approached members of the Kanker platform who indicated that they wanted to participate in research. We selected members with one of the following types of cancer: breast cancer, prostate cancer, gynecological cancer, lymphoma, colon cancer, rectal cancer, lung cancer, melanoma, or esophagus cancer (n=531). Kanker is the only web-based platform in the Netherlands for survivors of cancer and their relatives, where they can find trusted medical content and user-generated content. Kanker is an initiative of the Dutch Cancer Society (KWF Kankerbestrijding), the Dutch Federation of Cancer Patient Organisations, and the Netherlands Comprehensive Cancer Organisation. The platform started in 2013 with the functions of content, communication, and community. In 2020, Kanker had >500,000 visitors per month and approximately 32,500 members (July 2020). Members can make contact to communicate with other survivors and relatives, start a blog (1100 bloggers), participate in web-based discussion groups (50 groups), or participate in the research panel (1500 members). Visitors have to become members of Kanker for reading or posting user-generated content. The medical information is checked by professionals. To help the users generate content, Kanker has peer moderators.

The population-based group patients were asked by their physician to participate in the study and complete a questionnaire on paper. OCC members who indicated in their membership profile whether they were willing to complete questionnaires and who met our selection criteria (survivor and cancer type) were invited by the community manager of Kanker by email to participate in this study. This email contained a link to a web-based questionnaire.

As their names and addresses were unknown, a paper questionnaire could not be sent to the OCC group. The population-based group filled in an informed consent form before completing the questionnaire. Through an opt-in option in the Kanker terms of use, the OCC group gave their (informed) consent so that they could be approached to request their participation in the study.

### Measures

The NCR routinely collects data on tumor characteristics such as date of diagnosis; subsite; histology; stage (TNM clinical classification); primary treatment; and patient characteristics, including sex and date of birth. Kanker.nl respondents were asked to indicate certain tumor characteristics in the questionnaire (Multimedia Appendix 1 [15]; questionnaire translated; questions A, B, and C).

As no validated Dutch questionnaire on internet use among patients with cancer existed, we developed one in 2004, which was reviewed by an expert panel of 3 researchers and 6 survivors of cancer [27]. This questionnaire was based on the four areas of internet use—content, communication, community, and eHealth—defined by Eysenbach [28]. In 2017, we updated some of the questions because of internet developments in the intervening years, including increased access to Kanker, eHealth, social media, and blended care [10] (Multimedia Appendix 1; questionnaire translated; questions 27 and 29-42).

We used the same questionnaire for both groups; the population-based group filled out this questionnaire offline, and the OCC group did so on the web. The number of survivors in the population-based group on the web was unknown, and all the OCC group members were active on the web. In the questionnaire, we asked patients about their sociodemographics, internet use, sources of information for health care–related purposes, wishes regarding future internet use for health care–related purposes, self-management skills, and the effect of internet use on their health care consumption.

### Statistical Analysis

**Figure 2:**
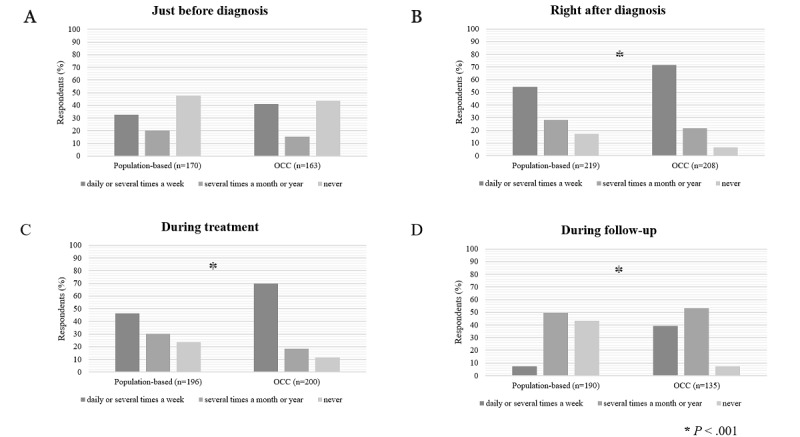
Internet search frequency for information on cancer just before diagnosis (A), right after diagnosis (B), during treatment (C), and during follow-up (D). OCC: online cancer community.

All statistical analyses were performed using the SPSS statistical software (version 24.0; IBM Corp). Data regarding patient characteristics were compared between the population-based and OCC groups using chi-square analyses for categorical variables and independent-sample, 2-tailed *t* tests for continuous variables (Table 1). Chi-square analyses were conducted to investigate differences between the population-based and OCC groups in (1) information sources found to be important (Table 2), (2) distributions of search frequencies for each different disease phase (Figure 2), and (3) effects of internet use and participation in OCCs (Multimedia Appendix 2, Table S1). Finally, separate multivariate logistic regression analyses were conducted to investigate the independent association between the type of population (population-based group vs OCC group) and internet search frequency (outcome) treated as a dichotomous variable (daily or several times a week vs several times a month or year, or never) while adjusting for patient (age, gender, and education) and disease (time since diagnosis) characteristics (Multimedia Appendix 2, Table S2). The tests were 2-sided, considered statistically significant at *P*<.05, and adjusted for multiple testing using the Bonferroni correction.

**Table 1:** Patient characteristics separated by type of patient group.

Characteristic			Netherlands Cancer Registry (population-based)		Kanker (OCC^a^)		*P* value	
Patients selected, N			523 (100)		531 (100)		N/A^b^	
Returned questionnaires^c^, n (%)			233 (44.6)		214 (40.3)		N/A	
**Gender, n (%)**								.66
	Female	142 (60.9)		126 (58.9)				
	Male	91 (39.1)		88 (41.1)				
**Age at time of survey (years), n (%)**								<.001
	<50	39 (16.7)		41 (19.2)				
	50-65	98 (42.1)		130 (60.7)				
	>65	96 (41.2)		43 (20.1)				
Age at time of survey (years), mean (SD)			61.8 (11.6)		58.1 (9.5)		<.001	
**Tumor, n (%)**								<.001
	Breast cancer	62 (26.6)		66 (30.8)				
	Prostate cancer	77 (33)		41 (19.2)				
	Gynecological cancer	64 (27.5)		17 (7.9)				
	Lymphoma	30 (12.9)		24 (11.2)				
	Other cancers^d^	N/A		66 (30.8)				
**Months since diagnosis, n (%)**								<.001
	0-18	6 (2.6)		51 (23.8)				
	19-24	50 (21.5)		12 (5.6)				
	25-30	74 (31.8)		15 (7)				
	31-42	103 (44.2)		135 (63.1)				
Months since diagnosis, median			29		42		<.001	
Months since diagnosis, mean (SD)			30.2 (6.9)		55.5 (59.0)		<.001	
**Education^e^, n (%)**								<.001
	Primary school	43 (18.6)		11 (5.1)				
	Secondary school	114 (49.4)		94 (43.9)				
	College or university	75 (32.5)		108 (50.5)				
**Employment status^f^, n (%)**								<.001
	Employed (ill)	94 (40.9)		124 (57.9)				
	Employed (on insurance)	25 (10.8)		70 (33.2)				
	Unemployed	136 (59.1)		90 (42.1)				
**Marital status^g^, n (%)**								.03
	Married or living together	191 (82)		174 (82.1)				
	Partner, not living together	13 (5.6)		3 (1.4)				
	No partner	28 (12)		36 (17)				
**Children^h^, n (%)**								.003
	None	33 (14.2)		50 (23.4)				
	Yes, living with one or both parents	40 (17.2)		51 (23.8)				
	Yes, living somewhere else	159 (68.5)		113 (52.8)				

^a^OCC: online cancer community.

^b^N/A: not applicable.

^c^Only internet users.

^d^Including colon cancer, rectal cancer, lung cancer, melanoma, esophagus cancer, and other.

^e^Missing for 2 patients.

^f^Missing for 3 patients.

^g^Missing for 2 patients.

^h^Missing for 1 patient.

**Table 2:** Sources of information found to be important (N=447).

Source of information	Ranking		Population-based (n=233), n (%)	OCC (n=214), n (%)	*P* value
	Population-based	OCC^a^			
Medical oncologist	1	1	212 (91)	189 (88.3)	.35
Oncology nurse	2	3	154 (66.1)	154 (72)	.71
Internet for information	3	2	126 (54.1)	171 (79.9)	<.001
Family	4	6	120 (51.5)	84 (39.2)	.009
Friends	5	7	115 (49.4)	76 (35.5)	.003
General practitioner	6	5	100 (42.9)	94 (43.9)	.83
Children	7	11	97 (41.6)	65 (30.4)	.01^b^
Other patients	8	4	60 (25.8)	107 (50)	<.001^b^
Other patients via the internet	15	10	17 (7.3)	68 (31.7)	<.001^b^
Group discussions with patients	17	8	13 (5.6)	70 (32.7)	<.001^b^
Colleagues	11	15	41 (17.6)	32 (15)	.45^b^
Pharmacist	9	14	48 (20.6)	40 (18.7)	.61^b^
Newspapers or television	9	12	49 (21)	63 (29.4)	.04
Books	11	8	41 (17.6)	71 (33.2)	<.001
Second-opinion physician	13	13	20 (8.6)	56 (26.2)	<.001
Alternative counselor	14	17	18 (7.7)	30 (14)	.03
Home care nurse	15	15	16 (6.9)	32 (15)	.006

^a^OCC: online cancer community.

^b^A relatively large difference in ranking (≥4).

## Results

### Overview

The two groups showed similar response rates: 47% (233/496) for the population-based group and 40.3% (214/531) for the OCC group. In the OCC group, 30.8% (66/214) had a cancer type other than lymphoma, prostate cancer, breast cancer, or gynecological cancer. As we found no significant differences between the results for patients with different cancer types, we only report the totals.

### Patient Characteristics

Differences between the 2 groups were evident with regard to patient characteristics (Table 1). Compared with the population-based group respondents, the OCC group respondents had a higher education level (college or university: 108/214, 50.5% vs 75/231, 32.5%; *P*<.001) and were younger (mean age 58.1, SD 9.5 years vs 61.8, SD 11.6 years; *P*<.001), and more respondents were employed (124/214, 57.9% vs 94/233, 40.3%; *P*<.001). Compared with the population-based group, the OCC group respondents had children less often (164/214, 76.6% vs 199/232, 85.7%; *P=*.003).

### Internet Use

The following results for questions about participation in an OCC were reported by the OCC and population-based group respondents, respectively: reading posts of other survivors (120/214, 56.1% vs 26/114, 22.8%; *P*<.001), creating a profile (158/214, 73.8% vs 16/114, 14%; *P*<.001), and actively posting text in a blog or a discussion group (35/214, 16.4% vs 6/114, 5.3%; *P*<.001; Multimedia Appendix 2, Table S3). Overall, the population-based group hardly participated in a web-based health community.

Regarding communication and social media known in 2017, the OCC and population-based group respondents mainly used email and WhatsApp to communicate about their illness with family members (148/214, 69.2% vs 149/233, 63.9%), children (101/214, 47.2% vs 123/233, 52.8%), friends (158/214, 73.8% vs 142/233, 60.9%), and their oncologist (73/214, 34.1% vs 61/233, 26.2%). Facebook and blog posts were used more often to communicate with fellow survivors (110/214, 51.4% vs 28/233, 12%). The other available media—Twitter and Skype—were rarely or never used. The OCC group reported more intensive use of digital media and maintained web-based contact with a greater variety of people (Multimedia Appendix 2, Table S4).

The OCC group respondents were less satisfied with the information they had received than the population-based group (131/214, 61.2% vs 200/233, 85.8%; *P*<.001). The OCC group attached greater importance to all information sources except family members and children (if any) than the population-based group. Most of the differences in the importance of the information sources were statistically significant, including the internet (171/214, 79.9% vs 126/233, 54.1%; *P*<.001), fellow survivors (107/214, 50% vs 60/233, 25.8%; *P*<.001), and mass media (63/214, 29.4% vs 49/233, 21%; *P*=.04; Table 2).

In their ranking of information sources on relative importance, there were many similarities between the population-based and OCC groups except for the importance that patients attached to fellow patients and their own children.

In almost all phases of the patient journey during the illness, the OCC group reported more intensive internet use (Figure 2). The differences in the three phases were significant: (1) immediately after diagnosis, (2) during treatment, and (3) during follow-up (*P*<.001 in each case). Only just before diagnosis, the distribution of internet use between the 2 groups did not differ significantly. These results were also found when adjusting for patient (age, gender, and education) and disease (time since diagnosis) characteristics (Multimedia Appendix 2, Table S2). In addition, the population-based group indicated *not applicable* more often in the *during treatment* phase, which suggests that the population-based group respondents received treatment less often, the difference being 16% (109/214, 50.9% vs 82/233, 35.2%). The population-based respondents reported being in the *follow-up* phase more often (189/233, 81.1% vs 135/214, 63.1%), which did not result in more intensive internet use in that phase. The population-based respondents were probably less seriously ill; thus, fewer treatments were needed to enter the follow-up phase. The OCC group underwent more treatments, and most are still in the treatment phase (Multimedia Appendix 2, Table S5).

In searching for information on all topics included in the questionnaire, the OCC group reported using the internet more intensively than the population-based group, the mean difference being 23%. Searching for information on *cancer support groups*, *trials/research*, and *type of cancer* diverged strongly from that mean (by 42%, 37%, and 13%, respectively). To determine whether both groups found the same topics important, the percentage for each group was used to rank the topics from 1 to 18. The 2 groups ranked nearly all topics equally on importance, except for *consequences for sexuality* (7 vs 12, respectively) and *cancer support groups* (13 vs 9, respectively; Multimedia Appendix 2, Table S6).

More survivors in the OCC group reported that after using the internet, they were better informed (92/214, 43% vs 68/233, 29.2%; *P=*.002) to discuss the information with their physician more often than the population-based group (21/214, 9.8% vs 9/233, 3.9%; *P=*.004). There were no differences in terms of whether the information they had obtained influenced their choice of treatment (45/214, 21% vs 37/233, 15.9%; *P*=.14). Neither group reported that their internet use led to more consultations with a physician (2/214, 0.9% and 2/233, 0.9%; Multimedia Appendix 2, Table S1).

### Wishes Regarding Internet Use

For all topics, survivors’ wishes with regard to internet use exceeded current possibilities (Table 3). The 2 groups reported similar use of resources on all topics. Their use at the time of completing the questionnaire differed by a mean of 5%, whereas the wishes regarding all topics differed by a mean of 16%.

For both groups, the difference between possibilities and wishes was greatest for *getting advice on supportive health care* (possibilities: 0%; wishes: 126/233, 54.1% and 148/214, 69.2%). The OCC group reported 24% higher wishes related to participating in a web-based self-management class and chatting with others (44/233, 18.9% vs 92/214, 43%).

**Table 3:** Patients’ current use of and future wishes for internet possibilities (N=447).

Item	Current use			Future wishes			Ranking wishes	
	Population-based (n=233), n (%)	OCC^a^ (n=214), n (%)	Population-based (n=233), n (%)		OCC (n=214), n (%)	Population-based		OCC
Accessing own test results	72 (30.9)	77 (36)	170 (73)		184 (86)	1		1
Accessing own medical file	75 (32.2)	77 (36)	165 (70.8)		182 (85)	2		2
Making an appointment	56 (24)	81 (37.9)	161 (69.1)		173 (80.8)	3		3
Requesting prescriptions	72 (30.9)	81 (37.9)	156 (67)		165 (77.1)	4		4
Getting personal advice on symptoms	N/A^b^	N/A	142 (60.9)		152 (71)	5		5
Emailing with oncologist	58 (24.9)	64 (29.9)	135 (57.9)		152 (71)	6		5
Getting advice on supportive care	N/A	N/A	126 (54.1)		148 (69.2)	7		7
Receiving reminders	56 (24)	56 (26.2)	123 (52.8)		143 (66.9)	8		9
Making complaints	58 (24.9)	88 (41.1)	123 (52.8)		139 (65)	8		11
Emailing with nurse	82 (35.2)	73 (34.1)	119 (51.1)		146 (68.2)	10		8
Self-monitoring of treatment consequences	N/A	N/A	112 (48.1)		143 (66.8)	11		9
Rating health care professionals or hospitals	N/A	N/A	93 (39.9)		116 (54.2)	12		14
Requesting tests	28 (12)	17 (7.9)	105 (45.1)		118 (55.1)	13		13
Suggesting ideas	35 (15)	47 (22)	96 (41.2)		139 (65)	14		11
Requesting referrals	21 (9)	28 (13)	96 (41.2)		111 (51.9)	14		15
Performing self-diagnosis tests	7 (3)	9 (4.2)	63 (27)		88 (41.1)	16		18
Participating in web-based self-management class	N/A	N/A	44 (18.9)		92 (43)	17		16
Requesting oncologist via forum	7 (3)	6 (2.8)	44 (18.9)		81 (37.9)	17		19
Chatting with others	9 (4)	11 (5.1)	42 (18)		92 (43)	19		16
Asking questions of an oncologist in forum	5 (2)	9 (4.2)	35 (15)		73 (34.1)	20		20

^a^OCC: online cancer community.

^b^N/A: not applicable.

## Discussion

### Principal Findings

Dutch survivors participating in a web-based cancer community (the OCC group) were younger, more educated, more likely to be employed, and more likely to be unemployed because of illness than the population-based group. Significantly fewer members of the OCC group had children, and they found fellow survivors and web-based group discussions relatively more important as sources of information than their close relatives.

### Differences in Patient Characteristics

Approximately 69.1% (148/214) of the OCC group were survivors of the same 4 cancer types as the survivors in the population-based group. The remaining 30.8% (66/214) were survivors of 6 other random cancer types. Our additional analyses demonstrated that information needs and internet use were not influenced by cancer type. This can be confirmed by previous studies that showed that information seeking and illness-coping styles seem to influence how patients process information [29,30].

To increase the reach among the average population, we decided to repeat our research method of 2005 and asked the population-based group to complete the questionnaires on paper. Importantly, earlier research has shown that there is no difference in response rate between different invitation modes [31,32]. In this study, we show that there are differences between the population-based and OCC groups, not only in terms of sociodemographic characteristics. The OCC group seemed to have undergone more treatments (Multimedia Appendix 2, Table S5). The OCC group may experience more late effects of their treatment and seem to have less control over the consequences of their disease and treatment. The active involvement in Kanker.nl suggests that they hope that change is still possible.

### Differences in Internet and Media Use

This study revealed significant differences in internet use between the population-based and OCC groups. The latter searched for information on clinical trials markedly more often. A possible explanation for this phenomenon, as indicated by previous studies, is that younger and highly educated respondents tend to search for such information more often and tend to understand it better than older respondents with a low level of education [33,34].

As far as we have been able to ascertain, only a limited portion (<25%) of the population-based group respondents participated in an OCC [10]. The OCC group found fellow survivors significantly more important as a source of information, which is probably why they participate in an OCC. Fellow survivors provide both emotional and informational support [15].

The OCC group respondents communicated more often with oncologists (73/214, 34.1% vs 61/233, 26.2%) and fellow survivors (110/214, 51.4% vs 28/233, 12%) than their population-based group counterparts and used more different media to interact with their social network in relation to their illness (Multimedia Appendix 2, Table S4).

These differences require not only access to information but also possession of health-related skills such as the ability to formulate meaningful questions [24,35,36]. Actively using the internet to access information, participate in an OCC, and communicate with their social network enables survivors to develop those skills [37]. Recent studies have shown that participating in such a community makes survivors more resilient, which also enables self-management [38,39].

### Differences in Wishes Regarding Internet Use

The 2 groups reported different wishes, although the ranking of the wishes in order of importance was markedly similar. This is in line with our previous study comparing internet use of survivors in the population-based group in 2005 and 2017 [10], which showed that the intensity of use changed with time, although the ranking of wishes remained stable.

Many of the survivors’ wishes were related to eHealth, which makes it possible for them to actively participate in illness and recovery management. An important aspect is access to their own electronic health record (EHR). According to the Netherlands’ eHealth monitor 2018, approximately 45% of citizens had access to their EHR [40]. This corresponds roughly to the use of their own medical file reported by OCC respondents. EHR use by the population-based respondents ranged from 24.9% (58/233) to 35.2% (82/233) in this study.

A possible explanation could be that the intensive internet users—in this study, the OCC group—are probably early adopters of eHealth. They would seem to be accurate indicators of future internet use by a large number of survivors [10]. If so, then in the coming years, eHealth interventions will be increasingly used to self-monitor one’s own illness management behavior. This effect may be amplified as more patients with cancer survive longer, often with more long-term and late effects.

### Differences in Treatment and Sense of Control

The OCC group underwent more treatments. It seems understandable that these survivors experience the consequences of treatment more and have an insufficient sense of control over these symptoms. The survivors actively searched for information and joined an OCC (Figure 2). The population-based group had fewer treatments and, therefore, fewer problems coping with their symptoms compared with the OCC group [41,42]. The OCC group had more reasons to investigate what could possibly help them, in which case eHealth tools for self-care are an accessible option [43].

The OCC group has the characteristics of patients with chronic disease [44,45]. For them, *patient-as-partner* is the most appropriate concept [24,25]. The more active attitude is confirmed in their more frequent internet searches on topics such as *trials/research*, *cancer support groups*, and *What can I do myself?* Within the possibilities, they also make greater use of eHealth and have more wishes for future active participation in their health situation, such as shared decision-making, monitoring side effects, and seeking personal advice. It is unclear whether the OCC group comprises survivors who less readily accept the consequences of their illness or are more aware of them or are less able to cope with them or expect their symptoms to diminish. They probably expect that they can improve their health through active participation and self-management. Indeed, the characteristics of the survivors in the OCC group are factors that influence the self-management of individuals in an eHealth environment [43]. Could this OCC group represent the starting point for user uptake and implementation of web-based interventions, many of which remain on the shelf [46,47]? It may be that eHealth feels too burdensome for survivors and that the interventions should be more focused on e-Learning. An example of this is the cancer support community in the United States [48], which is less stigmatizing and appeals to people’s motivation in combination with their abilities to learn and communicate. Follow-up research into the web-based wishes of OCC participants could be directed at determining to what extent this growing group of patients with chronic cancer is motivated to take a course through a web-based patient academy that appeals to people’s skills and possibilities.

### Limitations of the Study

This study has several limitations that need to be addressed. First, we approached and surveyed the 2 sample groups in different ways. The population-based group respondents were asked by a physician to participate in the study and completed a paper-based questionnaire, whereas the OCC group was invited to participate on the web through the Kanker platform. For the latter group, we knew neither who their physicians were nor where they lived. We could not send them a paper questionnaire, so they answered the questions on the web. Studies on the use of web-based questionnaires versus paper questionnaires show that these methods can be used side by side [31,49]. Although these different research methods are unlikely to cause differences in results, we are not sure whether our sample is fully representative of OCCs. It may be that the members included in this study were the more active users of the OCC. However, this active group will likely correspond to the group of survivors that researchers have access to.

Furthermore, the population-based group included a small group of respondents who actively participated in an OCC such as Kanker. We did not consider this as an exclusion criterion as in any population-based sample, there are survivors who participate in a web-based community. The differences between the 2 groups would have been larger if we had excluded these respondents.

A final limitation is that the study was conducted only in the Netherlands, where internet access is extremely high, and the respondents have an above-average education level. Although the typical Dutch survivor of cancer may be different in certain ways from those in other Western countries, previous studies have shown that there are many similarities between the web-based behavior of survivors in various countries [14-16,18].

### Conclusions

We conclude that survivors who participate in an OCC (both posters and lurkers) are not representative of survivors of cancer in general. There are significant differences in (1) sociodemographic characteristics, (2) internet use during their treatment journey, (3) internet search frequency during their cancer journey, and (4) participation wishes. However, there are also certain similarities in terms of the relative importance that survivors attach to the various information sources, the topics on which they seek information, and their wishes for future eHealth possibilities. Any differences in importance ranking can be attributed to the OCC group being an internet-based community that actively seeks contact with fellow survivors.

The above findings and conclusions have implications for other researchers. Most importantly, if they recruit study participants through an OCC, they will not be fully representative of the general patient population. Arguably, an OCC group is more suitable for research into supportive care in relation to survivorship. The survivors in the OCC group experience long-term effects and seem motivated to gain a sense of control over them, which could be a good motivational factor to participate in web-based intervention studies. In general, it is advisable to take the specific nature of an OCC sample into consideration when reporting findings for this particular group of survivors of cancer.

In general, we recommend that survivors of cancer use internet resources throughout their illness and treatment journey. There are differences between the 2 groups because of the circumstances in which they find themselves; however, the internet offers different options for different circumstances. The wishes are similar; however, the use differs, which could be explained by age, gender, number of treatments, and communication needs.

Web-based information and communication can support shared decision-making and may facilitate the active participation of patients during their treatment. At the start of that journey, they have a great need for information, which is essential for shared decision-making [36]. After cancer treatment, such a platform provides patients with chronic cancer with an environment that seems to facilitate their active participation in their treatment [24,39].

## Appendix

Multimedia Appendix 1 Questionnaire translated.

Multimedia Appendix 2 Tables with additional data.

## Abbreviations

EHR: electronic health record
NCR: Netherlands Cancer Registry
OCC: online cancer community
