# Supplementary material for: Comparing Survivors of Cancer in Population-Based Samples With Those in Online Cancer Communities: Cross-sectional Questionnaire Study
Source: JMIR Cancer. 2022 Mar 8;8(1):e19379. doi: 10.2196/19379 (PMC8941444; doi:10.2196/19379)
Supplement: Multimedia Appendix 1 [file cancer_v8i1e19379_app1.docx]

## **Multimedia Appendix 1. Questionnaire translated.**

## **Questionnaire Internet use, M.C. van Eenbergen, et al.**

Automatically translated Google translate; dutch-english

A. Enter the month and year of diagnosis here:

B. For which cancer type were you (last) treated

- - Cervical cancer
  - Uterine cancer
  - Breast cancer
  - Colon cancer
  - Ovarian cancer
  - Rectal cancer
- Lung cancer
  - Lymph node cancer
  - Melanoma
  - Prostate cancer
  - Otherwise, namely

C. What treatments have you had (more answers possible)

- Irradiation
- Chemotherapy
- Targeted therapy
- Hormone therapy
- Immunotherapy
- Operation
- Stem cell transplant
- Otherwise, namely

## General questions

1. Enter today's date here:

2. Enter your date of birth here:

3. What is your gender?

Man / Woman

4. What is your current civil status

- I am married
- I am not married, but I live with my partner
- I have a permanent partner, but we live separately
- I don't have a permanent partner

5. Do you have children (more answers possible)

- No I do not have any children
- Yes, I have one or more children living at home
- Yes, I have one or more children living away from home

6. Tick the highest level of education that you have completed

- None (or some primary education classes)
- Elementary School
- Vocational Education (LBO)
- Secondary General Secondary Education (such as MAVO, MULO)
- Secondary (vocational) education (MBO)
- Higher General Secondary Education (such as HAVO, Atheneum, Gymnasium)
- Higher Vocational Education (HBO)
- Scientific (university) education

7. We are curious about your current work situation? (more answers possible)

- I have work (paid / unpaid)
- I have a job, but make full use of the Sickness Benefits Act
- I have a job, but make partial use of the sickness benefit
- I am unemployed
- I am looking for a job
- I am a student
- I am a housewife / house husband
- I am incapacitated for work
- I am retired
- Otherwise, namely

### Information from the hospital

**The following questions are about how satisfied you are with the information you have received about your disease from various sources.**

8. Are you satisfied with the amount of information you received from the healthcare provider (s) about your illness? Not at all / A little bit / Quite badly / Very badly

9. How important are the following sources of information for you when it comes to your cancer disease? If the subjects mentioned are not applicable, do not tick anything. (4-point scale: 1 = very important / 4 = unimportant)

1. Your GP
2. Your treating specialist
3. The (oncology) nurse
4. Home care nurse
5. A second opinion doctor
6. Your pharmacy
7. Family members
8. Friends
9. Children
10. Colleagues
11. Alternative counselor
12. Fellow sufferers
13. Books
14. Newspapers / magazines / television
15. Internet for information
16. Discussion group on the Internet
17. Fellow group via social media

### General use of the Internet

**The following questions are about how you use the Internet in everyday life. These questions are therefore separate from the diagnosis of cancer.**

10. Which of the following means of communication do you use to visit the Internet? (more answers possible)

- Computer or laptop
- Smartphone
- Tablet / iPad
- Otherwise, namely
- No, I do not use the internet (continue with question #)

*If you have answered question 10 with NO, and therefore do not use the internet, you can stop filling in this questionnaire. You are done.*

*Thank you very much for filling in. Please kindly return the questionnaire*

11. Where do you use the internet? (more answers possible)

- At home (continue with question #)
- At work (continue with question #)
- Elsewhere, namely (continue with question #)
- Other people do the same for me

12. If you indicated in the previous question that other people also consult the Internet for you, who are they? (more answers possible)

- My partner
- My children
- Other family members
- Friends
- Colleagues
- Otherwise, namely

*Because others consult the internet for you, it is difficult for you to complete the remainder of this questionnaire. You can therefore stop filling in this questionnaire. You are done.*

*Thank you very much for filling in. Please kindly return the questionnaire*

13. How often do you use the internet on average

- Daily
- Once / several times a week
- Less than once a week
- Less than once a month
- Otherwise, namely

14. On average, how many hours do you spend privately on the Internet (including chatting, emailing, games, etc.)?

- More than 3 hours a day
- Between 2 and 3 hours a day
- Between 1 and 2 hours a day
- Between 3 and 7 hours a week
- Less than 1 hour a week

### Information on the Internet

**The Internet can be used in various ways, such as emailing, chatting, playing games, downloading music, shopping. The next set of questions is about the Internet as a source of information.**

15. How often did you search for information about cancer on the Internet? [Not all phases listed below may apply to you.]

*Check one box on each line: daily / one to three times p.wk / one to three times p.month / one to three times p.year / never / n.a.*

FOR DIAGNOSIS

1. When I had complaints, but did not yet know what it was
2. During the period that I was examined in the hospital, but did not know that there was cancer

DIAGNOSIS

1. After the specialist told that it was cancer

THERAPY

1. During the period that I had to wait for the operation / treatment
2. During chemotherapy / hormone therapy treatment
3. During radiotherapy treatment

AFTER TREATMENT (checks)

1. Since I am no longer treated, but still go to the doctor for checks

TREATMENT NOT (MORE) POSSIBLE (palliative phase)

1. Now that it's clear that I can't get any better

16. How did you search for information about cancer? (more answers possible)

- Using a search engine
- Directly to a specific website, of which I knew the address
- Using links on other websites
- Otherwise, namely

17. Have you searched online for information about healthcare abroad If so, on which subjects? (more answers possible)

- Not wanted
- Searched for expert cancer specialists
- Searched for treatment options
- Searched for the quality of hospitals
- Searched for ongoing experimental cancer research
- Searched for medicines
- Otherwise, namely

18. We would like to know if you have searched for information on the topics below and whether you have actually found this information?

*Tick ​​what corresponds to your situation (more than 1 cross on a line is possible): searched and found / searched and not found / not searched*

1. About your cancer
2. What treatments are possible
3. What the consequences of the treatment may be
4. What alternative medicine there are
5. Where to find a good doctor
6. What is the best hospital
7. What patient associations have to offer
8. What the activities are for patients in the region
9. Which trials / studies are running
10. What the treatment guidelines are
11. What are the options for contact with fellow sufferers
12. What you can do yourself
13. What the consequences are for your sexuality
14. How it is that you are so tired
15. About other symptoms during or after treatment
16. About additional care options (via the Internet)
17. About lifestyle and health
18. About cancer and heredity
19. How you can influence your end of life
20. What your health insurer reimburses
21. you. What are the financial consequences of your illness
22. About legal regulations (eg insurance)
23. About possibilities for future parenting
24. X. Otherwise, namely

### Effect of Internet use

**Has the information you have found on the internet also affected your personal well-being or the treatment you have received? This is what the following series of questions are about.**

19. In your opinion, were you better informed about your illness after consulting the information on the Internet?

- Yes, I was better informed
- Yes, I was better informed, but at the same time I received many new questions
- No difference
- No, I was not better informed, because I got a lot of new questions
- No, I was not better informed, because I could not judge the information for accuracy

20. Do you think that by visiting the internet, the visit to your doctor has increased

- Yes, that is why I went to my doctor more often
- It does not matter; I just visited my doctor according to the agreements already made
- No, I went less often
- I don't know if it made a difference

21. Have you discussed the information you have found on the internet with your care providers?

Never / Sometimes / Usually yes / Always / Different

22. Do you have the impression that the information found has influenced the choice of your doctor for your therapy / treatment?

Yes No

### What and where to search on the internet

**It is important for us to know where you expect to find the information about cancer on the internet, and which websites you regularly visit or have visited. The following questions include these expectations.**

23. Have you visited the following websites to find information about your cancer?

*Tick ​​one block on each line: daily / one to three times p.wk / one to three times p.mnd / one to three times p.year / never*

1. Website (s) of hospitals
2. Website (s) of universities
3. Website (s) of patient associations
4. Website (s) of scientific journals
5. Website (s) of health organizations (e.g. KWF Cancer Fighting)
6. Website (s) of other patients / fellow sufferers
7. Website (s) of doctors who provide advice via the internet
8. Website (s) of pharmaceutical manufacturers (pharmaceutical industry)
9. Website (s) of alternative care providers
10. Website (s) of companies that sell health products
11. Other websites, namely

24. Which websites would you most like to consult for reliable cancer information?

*The said care providers or organizations may not (yet) have information about cancer on their website. In that case, imagine they do. (Check one box on each line) 5-point scale: 1 = preferable / 5 = preferable not*

I would consult the website of:

1. My GP
2. My medical specialist (s)
3. My pharmacist
4. The hospital
5. The physician organizations (e.g. KNMG.nl)
6. The patient organizations / NFK
7. The health insurer
8. The pharmaceutical industry
9. Government
10. KWF Fight against cancer
11. Integrated Cancer Center of the Netherlands (IKNL)
12. An alternative counselor
13. Scientific journals
14. One website with all information about cancer
15. Other, namely.

25. What are your favorite Dutch websites if you are looking for information about cancer?

1. / 2. / 3. / 4. / 5.

26. What are your favorite foreign websites if you are looking for information about cancer

1. / 2. / 3. / 4. / 5.

### Internet as a contact option

**The previous questions were about searching for information via the internet. Another possibility is that you contact (unknown) acquaintances via the internet. The most famous form is sending e-mails. Other forms of contact are via discussion groups on websites or via social media such as Facebook, Twitter, WhatsApp. We would like to know from you whether you (have) used the internet to contact and / or maintain contact with fellow sufferers.**

27. Do you want to tick which of the following services you have an account with? (more answers possible)

- Facebook
- Twitter
- WhatsApp (or Telegram, etc.)
- LinkedIn
- Kanker.nl
- Skype (or Facetime, etc.)
- Lifestyle related apps on phone or tablet / iPad
- I do not have an account on (of the mentioned) social network site (s)
- I (also) have an account on another social network environment, namely:

28. Have you had the need during your illness period to ask one of the care providers below by e-mail? Check a box on each line: Yes / No.

- Your doctor
- Your treating specialist
- The (oncology) nurse
- Your pharmacy
- Another care provider, namely:

29. Was contact via the internet with the persons below important during your illness period?

*If not listed, check the option that most closely matches. If said persons were "not applicable", do not tick anything.*

Important: Yes / No / Not possible

How: Email / Face-book / Twitter / Whats-app / Blog / Skype or Facetime

1. Family members
2. Friends
3. Children
4. Colleagues
5. Your doctor
6. Your treating specialist
7. The (oncology) nurse
8. A second opinion doctor
9. Your pharmacy
10. An unknown (internet) doctor
11. An alternative counselor
12. Fellow sufferers
13. Members of a discussion group
14. Otherwise, namely:

30. Have you ever visited the website www.kanker.nl?

Yes / No (if no, continue with question 35)

31. How did you come to www.cancer.nl?

- Through my doctor
- Via a search engine, such as Google
- Through family / friends
- Otherwise, namely

32. How often did you visit cancer.nl in the past month?

- Daily
- Several times a day
- Once / several times a week
- Less than once a week
- Less than once this month
- Not

33. If you visit cancer.nl, what do you do? (more answers possible)

- I visit the cancer.nl library to look up information
- I have created a profile so that I can read everything
- I have created a profile so that I can post messages myself
- I have a blog
- I contact people who are the same as me
- Otherwise, namely

34. The information in the library of cancer.nl: (Check one of the boxes on each line)

1 = Strongly agree / 5 = Strongly disagree

1. I find it very understandable (I get it)
2. I find very complete (everything I want to know, I find there)
3. I find it objectively written (independent)
4. I find it easy to read (well written)
5. I think it is important to know

35. Have you ever "read along" with fellow sufferers during your illness period through a forum, blog, discussion platform or "community" of cancer patients?

- No.
- No, I want to but I can't find a suitable site
- Yes, via the following website (s):

36. Have you ever spoken via the internet (chat / facebook) to fellow sufferers?

- No.
- No, I want to but I can't find a suitable site
- Yes, via the following website (s):

37. Have you started a blog about your illness? (more answers possible)

- Yes, on cancer.nl
- Yes, on another website, namely:
- No.

38. Did you yourself post a comment in the last month about your illness? (more answers possible)

- Yes, on cancer.nl
- Yes, on another website, namely:
- No If no, continue with question 40

39. If you started a blog or participate in the discussion, how many messages did you write in the past month?

1 or 2/3 to 5/6 to 10 / more than 10

40. Have you created a profile on a website to share experiences with fellow sufferers? (more answers possible)

Yes, on cancer.nl / Yes, on another website, namely: / No.

41. What are your experiences with online forms of peer contact?

Positive / Negative / Not positive, not negative / Does not apply

42. How likely is it that you would recommend cancer.nl to family / friends / colleagues?

1 = very likely / 5 = not likely

### Use of the Internet for personal, medical information

**The Internet also offers the possibility of making personal, privacy-sensitive information accessible. A good example of this is internet banking. Healthcare makes little use of these options. The following questions are about your wishes to be able to follow your personal health situation via the internet.**

43. Do you currently have access to your medical file via the Internet? (Multiple answers possible)

- No (continue with question 47)
- Yes, in the medical file with the GP
- Yes, in the hospital's medical file
- Otherwise, namely

44. Do you take the opportunity to consult your medical file?

No / Yes

45. How do you feel about having access to your medical file via the internet?

Positive / Negative / Not positive, not negative

46. Assuming that your medical data is properly secured, would you like to be able to view it via the internet?

- Yes, I want to be able to view my medical data via the internet.
- Yes, I want to be able to view my medical data via the internet and decide for myself who has more access to my data (eg which doctor, pharmacy or other healthcare provider).
- Yes, I want to be able to view my medical data via the internet and to decide for myself who has more access to my data. In addition to social workers, I also want to give access to my immediate family so that they know better what is wrong with me.
- No, I don't have to.

47. If you could ask the opinion of another doctor (second opinion) via the Internet, would you do that?

No / Yes, but I haven't done that yet / Yes, and I have done that too / I don't know

48. What options do your doctor or hospital provide via the internet? (Check one box on each line}

Yes No

1. Being able to view own medical data
2. Ability to request results of examinations
3. Can e-mail with the treating physician (s)
4. Can e-mail with the nurses involved
5. Can request and / or extend recipes
6. Being able to request diagnostic research
7. Can request a referral to another specialist
8. Being able to make an appointment with your own doctors
9. Being able to do self-diagnosis tests
10. Being able to post a question my doctor answers through a forum (anonymous, but public to others)
11. Being able to post a question my doctor answers and others can respond to
12. Can "talk" to fellow sufferers who are treated for cancer in the same hospital
13. Being able to receive reminders to support treatment
14. Able to report complaints
15. Being able to come up with ideas for improving treatment
16. Otherwise, namely:

49. If your doctor or a health organization has a (secure) website, what would you use? Check one of the boxes on each line

1 = Very much like / 5 = Exactly not

1. Being able to view own medical data
2. Ability to request results of examinations
3. Can e-mail with the treating specialist
4. Can e-mail with the nurses involved
5. Can request and / or extend recipes
6. Being able to request diagnostic research
7. Can request a referral to another specialist
8. Being able to make an appointment with your own doctors
9. Being able to do self-diagnosis tests
10. Being able to post a question that my doctor answers through a forum (public for others)
11. Being able to post a question my doctor answers and others can respond to
12. Can "talk" (chat) with fellow sufferers who are treated in the same hospital for cancer
13. Being able to receive reminders to support treatment
14. Able to report complaints
15. Being able to come up with ideas for improving treatment
16. Able to keep track of things that bother you (such as fatigue)
17. Can receive personalized advice tailored to your symptoms
18. Being able to receive an overview of additional care options
19. Being able to assess a healthcare provider or healthcare institution
20. Can participate in an online self-help course
21. Otherwise, namely:

### Working together on health

**Below are a number of statements that say something about how you deal with your disease and its treatment. With each statement you can give your opinion by circling the number that best suits your situation.**

50. We would like to know something about how you deal with your health condition.

Check one of the boxes on each line; 0 = very little or not so good or never / 4 = a little or reasonable or sometimes / 8 = very much or very good or always

1. In general I know about my condition (s)
2. In general, I know about the treatment and the medications for my condition (s)
3. I use the medications and perform the treatments that my doctor or health care provider has suggested
4. I take decisions about (the treatment of) my condition (s) together with my doctor or healthcare provider
5. I am able to arrange with my care provider that I receive the care I need and that suits my culture, values ​​and views
6. I arrange the (follow-up) appointments that my doctor or healthcare provider proposes and also go there
7. I keep an eye on symptoms and signals from my body (such as blood sugar levels, weight, shortness of breath, pain, sleeping problems, mood)
8. I intervene myself when my body gives signals and symptoms get worse
9. I can deal with the effect of my condition (s) on my physical activities (such as walking, housework)
10. I can deal with the effect of my condition (s) on how I feel (ie my emotions and mental well-being)
11. I can deal with the consequences of my condition (s) for my social contacts (i.e. dealing with other people)
12. In general, I manage to live a healthy life (eg no smoking, moderate alcohol consumption, healthy eating, exercising regularly, coping with stress)

| 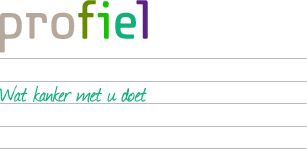  **DUTCH VERSION** |
| --- |

**Vragenlijst over internetgebruik**

Dit is een vragenlijst over het gebruik van internet voor mensen die in 2013, 2014 of 2015 zijn gediagnosticeerd met een vorm van kanker. Wij stellen het op prijs als u deze vragenlijst wilt invullen. Ook als u minder vaak gebruik maakt van internet, zien wij graag uw antwoorden tegemoet. Vul de vragenlijst zelf, op uw gemak, in.

U kunt steeds antwoord geven door het hokje/cijfer aan te kruisen dat het beste bij u past. Als u twijfelt, geef dan het antwoord dat het dichtst in de buurt komt van uw situatie. Er zijn geen goede of foute antwoorden; het gaat om uw persoonlijke mening of ervaring. Hoewel sommige vragen op elkaar kunnen lijken, is iedere vraag anders. Het kan ook zijn dat sommige vragen voor u overbodig lijken of niet van toepassing. Wilt u toch proberen alle vragen te beantwoorden?

De antwoorden op deze vragenlijst worden vertrouwelijk behandeld en zullen niet herleidbaar zijn naar personen, en alleen gebruikt worden voor dit onderzoek.

|  | Datum: | | | | | | | | | | | | |
| --- | --- | --- | --- | --- | --- | --- | --- | --- | --- | --- | --- | --- | --- |
|  | | | | | | | | | | | | | |
|  | |  |  | - |  |  | - |  |  |  |  |  | |
|  | | | | | | | | | | | | | |
|  | Studienummer: | | | | | | | | | | | | |
|  | | | | | | | | | | | | | |
|  | |  |  |  |  |  |  |  |  |  |  |  |  |
|  | | | | | | | | | | | | | |

Invulinstructie

⦁ Vul de vragenlijst in met een zwarte of blauwe pen (geen viltstift).

⦁ Zet een duidelijk kruisje in het antwoordvakje.

⦁ Als u een fout antwoord invult, laat dan het foutieve kruisje staan en maak het goede vakje **helemaal zwart.**

⦁ Vul bij een getal één cijfer per vakje in. Het hele cijfer moet binnen het vakje komen. Geen streepjes zetten als u iets niet hoeft in te vullen.

⦁ Kruis bij elke vraag één hokje aan. Als er bij een vraag meer antwoorden gegeven mogen worden staat dit aangegeven.

| **Algemene vragen** |
| --- |

| 1. | Vul hier de datum van vandaag in: |  |  | / |  |  | / | 2 | 0 |  |  |
| --- | --- | --- | --- | --- | --- | --- | --- | --- | --- | --- | --- |

| 2. | Vul hier uw geboortedatum in: |  |  | / |  |  | / | 1 | 9 |  |  |
| --- | --- | --- | --- | --- | --- | --- | --- | --- | --- | --- | --- |

| 3. | Wat is uw geslacht? |
| --- | --- |
|  |  |
| ❒ | Man |
| ❒ | Vrouw |

| 4. | Wat is op dit moment uw burgerlijke status? |
| --- | --- |
|  |  |
| ❒ | Ik ben gehuwd |
| ❒ | Ik ben niet gehuwd, maar woon wel samen met mijn partner |
| ❒ | Ik heb een vaste partner, maar wij wonen apart |
| ❒ | Ik heb geen vaste partner |

| 5. | Heeft u kinderen? *(meerdere antwoorden mogelijk)* |
| --- | --- |
|  |  |
| ❒ | Nee, ik heb geen kinderen |
| ❒ | Ja, ik heb één of meer thuiswonende kinderen |
| ❒ | Ja, ik heb één of meer uitwonende kinderen |

| 6. | Kruis aan wat het hoogste niveau van opleiding is dat u heeft afgerond? |
| --- | --- |
|  |  |
| ❒ | Geen (of enkele klassen lager onderwijs) |
| ❒ | Basisschool |
| ❒ | Lager Beroeps Onderwijs (LBO) |
| ❒ | Middelbaar Algemeen Voortgezet Onderwijs (zoals MAVO, MULO) |
| ❒ | Middelbaar (beroeps-) onderwijs (MBO) |
| ❒ | Hoger Algemeen Voortgezet Onderwijs (zoals HAVO, Atheneum, Gymnasium) |
| ❒ | Hoger Beroeps Onderwijs (HBO) |
| ❒ | Wetenschappelijk (universitair) onderwijs |

| 7. | We zijn benieuwd naar uw huidige werksituatie *(meerdere antwoorden mogelijk)* | |
| --- | --- | --- |
|  |  | |
| ❒ | Ik heb werk (betaald / onbetaald) | |
| ❒ | Ik heb werk, maar maak volledig gebruik van de ziektewet | |
| ❒ | Ik heb werk, maar maak gedeeltelijk gebruik van de ziektewet | |
| ❒ | Ik heb geen werk | |
| ❒ | Ik ben op zoek naar werk | |
| ❒ | Ik ben student | |
| ❒ | Ik ben huisvrouw / huisman | |
| ❒ | Ik ben arbeidsongeschikt | |
| ❒ | Ik ben gepensioneerd | |
| ❒ | Anders, namelijk |  |

| **Informatie vanuit het ziekenhuis** |
| --- |

De volgende vragen gaan over hoe tevreden u bent met de informatie die u heeft gekregen over uw ziekte vanuit verschillende bronnen.

|  | | **Helemaal niet** | **Een beetje** | **Nogal erg** | **Heel erg** |
| --- | --- | --- | --- | --- | --- |
| 8. | Bent u tevreden over de hoeveelheid informatie die u kreeg van de zorgverlener(s) over uw ziekte? | ❒ | ❒ | ❒ | ❒ |

| 9. | | Hoe belangrijk zijn de volgende informatiebronnen voor u (geweest) als het over uw ziekte kanker gaat?  Indien de genoemde onderwerpen niet van toepassing zijn, kruist u niks aan. | | | | | | | |
| --- | --- | --- | --- | --- | --- | --- | --- | --- | --- |
|  |  | | | | | | | | |
|  | | | **Heel belangrijk** | | |  |  | **Onbelangrijk** | |
|  | | |  | | 1 | 2 | 3 | 4 |  |
| 1. Uw huisarts | | | |  | ❒ | ❒ | ❒ | ❒ |  |
| 1. Uw behandelend specialist | | | |  | ❒ | ❒ | ❒ | ❒ |  |
| 1. De (oncologie) verpleegkundige | | | |  | ❒ | ❒ | ❒ | ❒ |  |
| 1. De verpleegkundige van de thuiszorg | | | |  | ❒ | ❒ | ❒ | ❒ |  |
| 1. Een second opinion arts | | | |  | ❒ | ❒ | ❒ | ❒ |  |
| 1. Uw apotheek | | | |  | ❒ | ❒ | ❒ | ❒ |  |
| 1. Familieleden | | | |  | ❒ | ❒ | ❒ | ❒ |  |
| 1. Vrienden | | | |  | ❒ | ❒ | ❒ | ❒ |  |
| 1. Kinderen | | | |  | ❒ | ❒ | ❒ | ❒ |  |
| 1. Collega’s | | | |  | ❒ | ❒ | ❒ | ❒ |  |
| 1. Alternatieve hulpverlener | | | |  | ❒ | ❒ | ❒ | ❒ |  |
| 1. Lotgenoten | | | |  | ❒ | ❒ | ❒ | ❒ |  |
| 1. Boeken | | | |  | ❒ | ❒ | ❒ | ❒ |  |
| 1. Kranten / tijdschriften / televisie | | | |  | ❒ | ❒ | ❒ | ❒ |  |
| 1. Internet voor informatie | | | |  | ❒ | ❒ | ❒ | ❒ |  |
| 1. Discussiegroep op internet | | | |  | ❒ | ❒ | ❒ | ❒ |  |
| 1. Lotgenotengroep via sociale media | | | |  | ❒ | ❒ | ❒ | ❒ |  |

| **Algemeen gebruik van internet** |
| --- |

De volgende vragen gaan over hoe u internet in het dagelijks leven gebruikt. Deze vragen staan dus los van de diagnose kanker.

| 10. | | Welke van de volgende communicatiemiddelen gebruikt u voor bezoek aan internet?  *(meerdere antwoorden mogelijk)* | |
| --- | --- | --- | --- |
|  |  | | |
| ❒ | | Computer of laptop | |
| ❒ | | Smartphone | |
| ❒ | | Tablet / iPad | |
| ❒ | | Anders, namelijk |  |
| ❒ | | Nee, ik maak geen gebruik van internet | |

| **Als u vraag 10 met NEE beantwoord heeft, en dus géén gebruik maakt van internet, kunt u stoppen met het invullen van deze vragenlijst. U bent klaar.**  **Hartelijk dank voor het invullen. Wilt u zo vriendelijk zijn de vragenlijst wel terug te sturen?** |
| --- |

| 11. | | Waar maakt u gebruik van internet? *(meerdere antwoorden mogelijk)* | |
| --- | --- | --- | --- |
|  |  | | |
| ❒ | | Thuis (ga verder met vraag 13) | |
| ❒ | | Op mijn werk (ga verder met vraag 13) | |
| ❒ | | Elders, te weten | (ga verder met vraag 13) |
| ❒ | | Andere mensen doen dat voor mij | |

| 12. | | Als u bij de vorige vraag heeft aangegeven dat andere mensen internet voor u raadplegen, wie zijn dat dan? *(meerdere antwoorden mogelijk)* | |
| --- | --- | --- | --- |
|  |  | | |
| ❒ | | Mijn partner | |
| ❒ | | Mijn kinderen | |
| ❒ | | Andere familieleden | |
| ❒ | | Vrienden | |
| ❒ | | Collega’s | |
| ❒ | | Anders, namelijk |  |

| **Omdat anderen voor u internet raadplegen, is het lastig voor u om het vervolg van deze vragenlijst in te vullen. U kunt daarom stoppen met het invullen van deze vragenlijst. U bent klaar.**  **Hartelijk dank voor het invullen. Wilt u zo vriendelijk zijn de vragenlijst wel terug te sturen?** |
| --- |

| 13. | | Hoe vaak maakt u gemiddeld gebruik van internet? | |
| --- | --- | --- | --- |
|  |  | | |
| ❒ | | Dagelijks | |
| ❒ | | Eén / enkele keren per week | |
| ❒ | | Minder dan één keer per week | |
| ❒ | | Minder dan één keer per maand | |
| ❒ | | Anders, namelijk |  |

| 14. | | Hoeveel uren besteedt u gemiddeld privé op het internet (inclusief chatten, e-mailen, spelletjes, etc.) |
| --- | --- | --- |
|  |  | |
| ❒ | | Meer dan 3 uur per dag |
| ❒ | | Tussen de 2 en 3 uur per dag |
| ❒ | | Tussen de 1 en 2 uur per dag |
| ❒ | | Tussen de 3 en 7 uur per week |
| ❒ | | Minder dan 1 uur per week |

| **Informatie op Internet** |
| --- |

Internet kan op verschillende manieren worden gebruikt, zoals e-mailen, chatten, spelen van spelletjes, downloaden van muziek, winkelen.

De volgende reeks vragen gaat over internet als bron van INFORMATIE.

| 15. | Hoe vaak ging u op zoek naar informatie over kanker op internet?  [Niet alle fasen die hierna benoemd zijn, hoeven voor u van toepassing te zijn.] | | | | | | | | | | |
| --- | --- | --- | --- | --- | --- | --- | --- | --- | --- | --- | --- |
|  |  | | | | | | | | | | |
|  | | **1 = dagelijks**  **2 = één tot drie keer per week** | | | | | | | | |  |
|  | | **3 = één tot drie keer per maand** | | | | | | | | |  |
|  | | **4 = één tot drie keer per jaar** | | | | | | | | |  |
|  | | **5 = nooit** | | | | | | | | |  |
|  | | **6 = n.v.t.** | | | | | | | | |  |
|  | | |  | | | | | | | | |
| *Kruis één blokje aan op elke regel* | | |  |  |  |  |  | |  | | |
| **VOOR DIAGNOSE** | | | | **1** | **2** | **3** | **4** | **5** | | **6** |  |
| 1. Toen ik klachten had, maar nog niet wist wat het was | | | | ❒ | ❒ | ❒ | ❒ | ❒ | | ❒ |  |
| 1. Tijdens de periode dat ik onderzocht werd in het ziekenhuis, maar nog niet wist dat er sprake was van kanker | | | | ❒ | ❒ | ❒ | ❒ | ❒ | | ❒ |  |
| **DIAGNOSE** | | | | **1** | **2** | **3** | **4** | **5** | | **6** |  |
| 1. Nadat de specialist verteld had dat het om kanker ging | | | | ❒ | ❒ | ❒ | ❒ | ❒ | | ❒ |  |
| **BEHANDELING** | | | | **1** | **2** | **3** | **4** | **5** | | **6** |  |
| 1. In de periode dat ik moest wachten op de operatie/behandeling | | | | ❒ | ❒ | ❒ | ❒ | ❒ | | ❒ |  |
| 1. Tijdens de behandeling met chemotherapie/hormoontherapie | | | | ❒ | ❒ | ❒ | ❒ | ❒ | | ❒ |  |
| 1. Tijdens de behandeling met radiotherapie | | | | ❒ | ❒ | ❒ | ❒ | ❒ | | ❒ |  |
| **NA DE BEHANDELING** (controles) | | | | **1** | **2** | **3** | **4** | **5** | | **6** |  |
| 1. Sinds ik niet meer behandeld word, maar nog wel voor controles naar de arts ga | | | | ❒ | ❒ | ❒ | ❒ | ❒ | | ❒ |  |
| **BEHANDELING NIET (MEER) MOGELIJK** (palliatieve fase) | | | | **1** | **2** | **3** | **4** | **5** | | **6** |  |
| 1. Nu het duidelijk is dat ik niet meer beter kan worden | | | | ❒ | ❒ | ❒ | ❒ | ❒ | | ❒ |  |

| 16. | | Hoe heeft u gezocht naar informatie over kanker? *(meerdere antwoorden mogelijk)* | |
| --- | --- | --- | --- |
|  |  | | |
| ❒ | | Met behulp van een zoekmachine | |
| ❒ | | Direct naar specifieke website toe, waarvan ik het adres wist | |
| ❒ | | Met behulp van links op andere websites | |
| ❒ | | Anders, namelijk |  |

| 17. | | Heeft u via internet gezocht naar informatie over gezondheidszorg in het buitenland? Zo ja, over welke onderwerpen? *(meerdere antwoorden mogelijk)* | |
| --- | --- | --- | --- |
|  |  | | |
| ❒ | | Niet gezocht | |
| ❒ | | Gezocht naar deskundige kankerspecialisten | |
| ❒ | | Gezocht naar behandelingsmogelijkheden | |
| ❒ | | Gezocht naar de kwaliteit van de ziekenhuizen | |
| ❒ | | Gezocht naar lopend experimenteel kankeronderzoek | |
| ❒ | | Gezocht naar geneesmiddelen | |
| ❒ | | Anders, namelijk |  |

| 18. | | We zouden graag willen weten of u informatie heeft gezocht over onderstaande onderwerpen en of u deze informatie ook daadwerkelijk heeft gevonden. | | | | |
| --- | --- | --- | --- | --- | --- | --- |
|  |  | | | | | |
| *Kruis aan wat overeenkomt met uw situatie* | | | | **Gezocht en gevonden** | **Gezocht en**  **niet gevonden** | **Niet**  **gezocht** |
|  | | | |  |  |  |
| 1. Over uw vorm van kanker | | | | ❒ | ❒ | ❒ |
| 1. Wat voor behandelingen er mogelijk zijn | | | | ❒ | ❒ | ❒ |
| 1. Wat de gevolgen van de behandeling kunnen zijn | | | | ❒ | ❒ | ❒ |
| 1. Wat voor alternatieve geneeswijzen er zijn | | | | ❒ | ❒ | ❒ |
| 1. Waar een goede arts te vinden is | | | | ❒ | ❒ | ❒ |
| 1. Wat het beste ziekenhuis is | | | | ❒ | ❒ | ❒ |
| 1. Wat patiëntenverenigingen te bieden hebben | | | | ❒ | ❒ | ❒ |
| 1. Wat de activiteiten zijn voor patiënten in de regio | | | | ❒ | ❒ | ❒ |
| 1. Welke trials/onderzoeken er lopen | | | | ❒ | ❒ | ❒ |
| 1. Wat de behandelrichtlijnen zijn | | | | ❒ | ❒ | ❒ |
| 1. Wat de mogelijkheden zijn voor lotgenotencontact | | | | ❒ | ❒ | ❒ |
| 1. Wat u zelf kunt doen | | | | ❒ | ❒ | ❒ |
| 1. Wat de consequenties zijn voor uw seksualiteit | | | | ❒ | ❒ | ❒ |
| 1. Hoe het komt dat u zo moe bent | | | | ❒ | ❒ | ❒ |
| 1. Over andere symptomen tijdens of na behandeling | | | | ❒ | ❒ | ❒ |
| 1. Over aanvullende zorgmogelijkheden (via internet) | | | | ❒ | ❒ | ❒ |
| 1. Over leefstijl en gezondheid | | | | ❒ | ❒ | ❒ |
| 1. Over kanker en erfelijkheid | | | | ❒ | ❒ | ❒ |
| 1. Hoe u invloed kunt hebben op uw levenseinde | | | | ❒ | ❒ | ❒ |
| 1. Wat uw zorgverzekeraar vergoedt | | | | ❒ | ❒ | ❒ |
| 1. Wat de financiële gevolgen zijn van uw ziekte | | | | ❒ | ❒ | ❒ |
| 1. Over wettelijke regelingen (bijv. verzekeringen) | | | | ❒ | ❒ | ❒ |
| 1. Over mogelijkheden voor toekomstig ouderschap | | | | ❒ | ❒ | ❒ |
| 1. Anders, namelijk | | |  |  |  |  |

| **Effect van gebruik van internet** |
| --- |

Heeft de informatie die u op internet hebt gevonden ook gevolgen gehad voor uw persoonlijk welbevinden of voor de behandeling die u heeft gekregen? Daarover gaat de volgende reeks vragen.

| 19. | | Was u naar uw gevoel beter geïnformeerd over uw ziekte na het raadplegen van de informatie op internet? |
| --- | --- | --- |
|  |  | |
|  | |  |
| ❒ | | Ja, ik was beter geïnformeerd |
| ❒ | | Ja, ik was beter geïnformeerd, maar tegelijkertijd heb ik veel nieuwe vragen gekregen |
| ❒ | | Geen verschil |
| ❒ | | Nee, ik was niet beter geïnformeerd, want ik kreeg veel nieuwe vragen |
| ❒ | | Nee, ik was niet beter geïnformeerd, want ik kon de informatie niet beoordelen op juistheid |

| 20. | | Denkt u dat door het raadplegen van internet het bezoek aan uw arts is toegenomen? |
| --- | --- | --- |
|  |  | |
|  | |  |
| ❒ | | Ja, ik ben daardoor vaker naar mijn arts gegaan |
| ❒ | | Het maakt niet uit; ik heb gewoon volgens de reeds gemaakte afspraken mijn arts bezocht |
| ❒ | | Nee, ik ben minder vaak gegaan |
| ❒ | | Ik weet niet of het verschil heeft gemaakt |

| 21. | | Heeft u de informatie die u op internet heeft gevonden, met uw hulpverleners besproken? | |
| --- | --- | --- | --- |
|  |  | | |
| ❒ | | Nooit | |
| ❒ | | Soms | |
| ❒ | | Meestal wel | |
| ❒ | | Altijd | |
| ❒ | | Anders |  |

| 22. | | Heeft u de indruk dat de gevonden informatie van invloed is geweest op de keuze van uw arts voor uw therapie / behandeling? |
| --- | --- | --- |
|  |  | |
| ❒ | | Ja |
| ❒ | | Nee |

| **Wat en waar zoeken op internet** |
| --- |

Het is voor ons belangrijk om te weten waar u de informatie op internet over kanker verwacht(te) te vinden, en welke websites u regelmatig bezoekt of heeft bezocht. De volgende vragen gaan onder andere over deze verwachtingen.

| 23. | | Heeft u de volgende websites bezocht voor het vinden van informatie over uw kanker? | | | | | | | | | |
| --- | --- | --- | --- | --- | --- | --- | --- | --- | --- | --- | --- |
|  | | | | **1 = dagelijks** | | | | | | |  |
|  | | | | **2 = één tot drie keer per week** | | | | | | |  |
|  | | | | **3 = één tot drie keer per maand** | | | | | | |  |
|  | | | | **4 = één tot drie keer per jaar** | | | | | | |  |
|  | | | | **5 = nooit** | | | | | | |  |
|  | | | | |  | |  |  |  |  |  |
| *Kruis één blokje aan op elke regel* | | | | | **1** | | **2** | **3** | **4** | **5** |  |
| 1. Website(s) van ziekenhuizen | | | | | ❒ | ❒ | | ❒ | ❒ | ❒ |  |
| 1. Website(s) van universiteiten | | | | | ❒ | ❒ | | ❒ | ❒ | ❒ |  |
| 1. Website(s) van patiëntenverenigingen | | | | | ❒ | ❒ | | ❒ | ❒ | ❒ |  |
| 1. Website(s) van wetenschappelijke tijdschriften | | | | | ❒ | ❒ | | ❒ | ❒ | ❒ |  |
| 1. Website(s) van gezondheidsorganisaties (b.v. KWF Kankerbestrijding) | | | | | ❒ | ❒ | | ❒ | ❒ | ❒ |  |
| 1. Website(s) van andere patiënten/lotgenoten | | | | | ❒ | ❒ | | ❒ | ❒ | ❒ |  |
| 1. Website(s) van artsen die advies geven via internet | | | | | ❒ | ❒ | | ❒ | ❒ | ❒ |  |
| 1. Website(s) van geneesmiddelenproducenten (farmaceutische industrie) | | | | | ❒ | ❒ | | ❒ | ❒ | ❒ |  |
| 1. Website(s) van alternatieve hulpverleners | | | | | ❒ | ❒ | | ❒ | ❒ | ❒ |  |
| 1. Website(s) van bedrijven die gezondheidsproducten verkopen | | | | | ❒ | ❒ | | ❒ | ❒ | ❒ |  |
|  | Andere websites, n.l. | |  | | | | | | | |  |

| 24. | Welke websites zou u het liefste willen raadplegen voor betrouwbare informatie over kanker? De genoemde hulpverleners of organisaties hebben misschien (nog) geen informatie over kanker op hun website. In dat geval stelt u zich voor dat ze dat wel hebben. | | | | | | | | |
| --- | --- | --- | --- | --- | --- | --- | --- | --- | --- |
|  | | |  | |  |  |  |  | |
| *Kruis één hokje aan op elke regel* | | | **liever wel** | |  |  |  | **liever niet** | |
| **Ik zou de website raadplegen van**: | | |  | 1 | 2 | 3 | 4 | 5 |  |
| 1. Mijn huisarts | | | | ❒ | ❒ | ❒ | ❒ | ❒ |  |
| 1. Mijn medisch specialist(en) | | | | ❒ | ❒ | ❒ | ❒ | ❒ |  |
| 1. Mijn apotheker | | | | ❒ | ❒ | ❒ | ❒ | ❒ |  |
| 1. Het ziekenhuis | | | | ❒ | ❒ | ❒ | ❒ | ❒ |  |
| 1. De artsenorganisaties (b.v. KNMG.nl) | | | | ❒ | ❒ | ❒ | ❒ | ❒ |  |
| 1. De patiëntenorganisaties / NFK | | | | ❒ | ❒ | ❒ | ❒ | ❒ |  |
| 1. De zorgverzekeraar | | | | ❒ | ❒ | ❒ | ❒ | ❒ |  |
| 1. De farmaceutische industrie | | | | ❒ | ❒ | ❒ | ❒ | ❒ |  |
| 1. De overheid | | | | ❒ | ❒ | ❒ | ❒ | ❒ |  |
| 1. KWF Kankerbestrijding | | | | ❒ | ❒ | ❒ | ❒ | ❒ |  |
| 1. Integraal Kankercentrum Nederland | | | | ❒ | ❒ | ❒ | ❒ | ❒ |  |
| 1. Een alternatieve hulpverlener | | | | ❒ | ❒ | ❒ | ❒ | ❒ |  |
| 1. Wetenschappelijke tijdschriften | | | | ❒ | ❒ | ❒ | ❒ | ❒ |  |
| 1. Eén website met álle informatie over kanker | | | | ❒ | ❒ | ❒ | ❒ | ❒ |  |
|  | Anders, namelijk. |  | | | | | | | |

| 25. | Wat zijn uw favoriete Nederlandse websites als u informatie over kanker zoekt? | |
| --- | --- | --- |
|  | |  |
|  | 1. | |
|  | 2. | |
|  | 3. | |
|  | 4. | |
|  | 5. | |

| 26. | | Wat zijn uw favoriete buitenlandse websites als u informatie over kanker zoekt? |
| --- | --- | --- |
|  |  | |
|  | | 1. |
|  | | 2. |
|  | | 3. |
|  | | 4. |
|  | | 5. |

| **Internet als contactmogelijkheid** |
| --- |

De voorgaande vragen gingen over het zoeken naar informatie via internet. Een andere mogelijkheid is dat u contact zoekt met (on)bekenden via internet. De meest bekende vorm is het versturen van e-mails. Andere vormen van contact zijn via discussiegroepen op websites of via sociale media als Facebook, Twitter, WhatsApp. We willen graag van u weten of u internet (heeft) gebruikt om contact te zoeken en/of te onderhouden met lotgenoten.

| 27. | | Wilt u aankruisen bij welke van de volgende diensten u een account heeft?  *(meerdere antwoorden mogelijk)* | |
| --- | --- | --- | --- |
|  |  | | |
| ❒ | | Facebook | |
| ❒ | | Twitter | |
| ❒ | | WhatsApp (of Telegram, e.d.) | |
| ❒ | | LinkedIn | |
| ❒ | | Kanker.nl | |
| ❒ | | Skype (of Facetime e.d.) | |
| ❒ | | Leefstijlgerelateerde app’s op telefoon of tablet / iPad | |
| ❒ | | Ik heb géén account op een (van de genoemde) sociale netwerksite(s) | |
| ❒ | | Ik heb (ook) een account op een andere sociale netwerkomgeving, namelijk: | |
|  | |  |  |

| 28. | | Heeft u de tijdens uw ziekteperiode behoefte gehad om aan een van onderstaande hulpverleners een vraag via e-mail te stellen? | | | | | |  |
| --- | --- | --- | --- | --- | --- | --- | --- | --- |
|  |  | | |  | |  | |  |
|  | | *Kruis een blokje aan op elke regel* | Ja | | Nee | |  |  |
|  | | Uw huisarts | ❒ | | ❒ | |  |  |
|  | | Uw behandelend specialist | ❒ | | ❒ | |  |  |
|  | | De (oncologie)verpleegkundige | ❒ | | ❒ | |  |  |
|  | | Uw apotheek | ❒ | | ❒ | |  |  |
|  | | Een andere hulpverlener, namelijk: | ❒ | | ❒ | |  |  |
|  | |  | | | | | | |

| 29. | Was contact via internet met onderstaande personen belangrijk tijdens uw ziekteperiode? Indien niet genoemd, kruis dan de optie aan die het meest overeenkomt.  Indien genoemde personen ‘niet van toepassing’ waren, dan niks aankruisen. | | | | | | | | | | | | | |
| --- | --- | --- | --- | --- | --- | --- | --- | --- | --- | --- | --- | --- | --- | --- |
|  | *Kruis één hokje aan op elke regel* | | | | | | | | | | |  | | |
|  | | | **Belangrijk?** | | | **Hoe? Via:** | | | | | | | |  |
|  | | **Ja** | | **Nee** | **Niet mogelijk** | **E-mail** | **Face-book** | **Twit-ter** | **Whats-app** | **Blog** | **Skype/**  **Facetime** | |  |  |
| 1. Familieleden | | ❒ | | ❒ | ❒ | ❒ | ❒ | ❒ | ❒ | ❒ | ❒ | |  |  |
| 1. Vrienden | | ❒ | | ❒ | ❒ | ❒ | ❒ | ❒ | ❒ | ❒ | ❒ | |  |  |
| 1. Kinderen | | ❒ | | ❒ | ❒ | ❒ | ❒ | ❒ | ❒ | ❒ | ❒ | |  |  |
| 1. Collega's | | ❒ | | ❒ | ❒ | ❒ | ❒ | ❒ | ❒ | ❒ | ❒ | |  |  |
| 1. Uw huisarts | | ❒ | | ❒ | ❒ | ❒ | ❒ | ❒ | ❒ | ❒ | ❒ | |  |  |
| 1. Uw behandelend specialist | | ❒ | | ❒ | ❒ | ❒ | ❒ | ❒ | ❒ | ❒ | ❒ | |  |  |
| 1. De (oncologie)-verpleegkundige | | ❒ | | ❒ | ❒ | ❒ | ❒ | ❒ | ❒ | ❒ | ❒ | |  |  |
| 1. Een second   opinion arts | | ❒ | | ❒ | ❒ | ❒ | ❒ | ❒ | ❒ | ❒ | ❒ | |  |  |
| 1. Uw apotheek | | ❒ | | ❒ | ❒ | ❒ | ❒ | ❒ | ❒ | ❒ | ❒ | |  |  |
| 1. Een onbekende (internet)dokter | | ❒ | | ❒ | ❒ | ❒ | ❒ | ❒ | ❒ | ❒ | ❒ | |  |  |
| 1. Een alternatieve hulpverlener | | ❒ | | ❒ | ❒ | ❒ | ❒ | ❒ | ❒ | ❒ | ❒ | |  |  |
| 1. Lotgenoten | | ❒ | | ❒ | ❒ | ❒ | ❒ | ❒ | ❒ | ❒ | ❒ | |  |  |
| 1. Leden van een discussiegroep | | ❒ | | ❒ | ❒ | ❒ | ❒ | ❒ | ❒ | ❒ | ❒ | |  |  |
| 1. Anders, namelijk: | | |  | | | | | | | | | | | |

| 30. | | Heeft u de website [www.kanker.nl](http://www.kanker.nl) wel eens bezocht? |
| --- | --- | --- |
|  |  | |
| ❒ | | Ja |
| ❒ | | Nee *(indien nee, ga verder met vraag 35)* |

| 31. | | Hoe kwam u terecht op www.kanker.nl? | |
| --- | --- | --- | --- |
|  |  | | |
| ❒ | | Via mijn arts | |
| ❒ | | Via een zoekmachine, bijvoorbeeld Google | |
| ❒ | | Via familie / vrienden | |
| ❒ | | Anders, namelijk |  |

| 32. | | Hoe vaak heeft u de afgelopen maand kanker.nl bezocht? |
| --- | --- | --- |
|  |  | |
| ❒ | | Dagelijks |
| ❒ | | Enkele keren per dag |
| ❒ | | Een / enkele keren per week |
| ❒ | | Minder dan één keer per week |
| ❒ | | Minder dan één keer deze maand |
| ❒ | | Niet |

| 33. | | Als u kanker.nl bezoekt, wat doet u dan? *(meerdere antwoorden mogelijk)* | |
| --- | --- | --- | --- |
|  |  | | |
| ❒ | | Ik bezoek de bibliotheek van kanker.nl om informatie op te zoeken | |
| ❒ | | Ik heb een profiel aangemaakt, zodat ik alles kan lezen | |
| ❒ | | Ik heb een profiel aangemaakt, zodat ik zelf berichten kan plaatsen | |
| ❒ | | Ik heb een blog | |
| ❒ | | Ik zoek contact met mensen die hetzelfde hebben als ik | |
| ❒ | | Anders, namelijk |  |

| 34. | | De informatie in de bibliotheek van kanker.nl: | | | | | | | |  |
| --- | --- | --- | --- | --- | --- | --- | --- | --- | --- | --- |
|  |  | | | | | | | | |  |
|  | | | **Helemaal mee eens** | |  |  |  | **Helemaal mee oneens** | | |
| *Kruis één van de hokjes aan op elke regel* | | | | 1 | 2 | 3 | 4 | 5 |  | |
| 1. Vind ik heel begrijpelijk (ik snap het) | | | | ❒ | ❒ | ❒ | ❒ | ❒ |  | |
| 1. Vind ik heel volledig (alles wat ik wil weten, vind ik er) | | | | ❒ | ❒ | ❒ | ❒ | ❒ |  | |
| 1. Vind ik objectief geschreven (onafhankelijk) | | | | ❒ | ❒ | ❒ | ❒ | ❒ |  | |
| 1. Vind ik prettig leesbaar (is goed geschreven) | | | | ❒ | ❒ | ❒ | ❒ | ❒ |  | |
| 1. Vind ik belangrijk om te weten | | | | ❒ | ❒ | ❒ | ❒ | ❒ |  | |

| 35. | Heeft u tijdens uw ziekteperiode wel eens ‘meegelezen’ met lotgenoten via een forum, blog, discussieplatform of ‘community’ van patiënten met kanker? | |
| --- | --- | --- |
|  |  | |
| ❒ | Nee | |
| ❒ | Nee, ik wil wel maar ik kan geen geschikte site vinden | |
| ❒ | Ja, via de volgende website(s): |  |

| 36. | Heeft u via het internet wel eens gesproken (chat / facebook) met lotgenoten? | |
| --- | --- | --- |
|  |  | |
| ❒ | Nee | |
| ❒ | Nee, ik wil wel maar ik kan geen geschikte site vinden | |
| ❒ | Ja, via de volgende website(s): |  |

| 37. | | Bent u een blog gestart over uw ziekte? *(meerdere antwoorden mogelijk)* | |
| --- | --- | --- | --- |
|  |  | | |
| ❒ | | Nee | |
| ❒ | | Ja, op kanker.nl | |
| ❒ | | Ja, op een andere website, namelijk: |  |

| 38. | | Heeft u de afgelopen maand zelf een reactie geplaatst in een discussiegroep over uw ziekte?  *(meerdere antwoorden mogelijk)* | |
| --- | --- | --- | --- |
|  |  | | |
| ❒ | | Ja, op kanker.nl | |
| ❒ | | Ja, op een andere website, namelijk: |  |
| ❒ | | Nee | |

| 39. | | Indien u met een blog gestart bent of meediscussieert, hoeveel berichten heeft u de afgelopen maand geschreven? |
| --- | --- | --- |
|  |  | |
| ❒ | | 1 of 2 |
| ❒ | | 3 tot 5 |
| ❒ | | 6 tot 10 |
| ❒ | | meer dan 10 |
| ❒ | | n.v.t. |

| 40. | | Heeft u een profiel aangemaakt op een website om ervaringen te kunnen delen met lotgenoten? *(meerdere antwoorden mogelijk)* | |
| --- | --- | --- | --- |
|  |  | | |
| ❒ | | Ja, op kanker.nl | |
| ❒ | | Ja, op een andere website, namelijk: |  |
| ❒ | | Nee | |

| 41. | Wat zijn uw ervaringen met online vormen van lotgenotencontact? |
| --- | --- |
|  |  |
| ❒ | Positief |
| ❒ | Negatief |
| ❒ | Niet positief, niet negatief |
| ❒ | Niet van toepassing |

| 42. | | Hoe waarschijnlijk is het dat u kanker.nl zou aanraden aan familie / vrienden / collega’s? | | | | | | |
| --- | --- | --- | --- | --- | --- | --- | --- | --- |
|  |  | | | | | | | |
| Heel waarschijnlijk | | | 1 | 2 | 3 | 4 | 5 | niet waarschijnlijk |
|  | | | ❒ | ❒ | ❒ | ❒ | ❒ |  |

| **Gebruik van internet voor persoonlijke, medische informatie** |
| --- |

Internet biedt ook de mogelijkheid om persoonlijke, privacygevoelige informatie toegankelijk te maken. Een goed voorbeeld daarvan is internetbankieren. De gezondheidszorg maakt nog maar weinig gebruik van deze mogelijkheden. De volgende vragen gaan over uw wensen om via internet uw persoonlijke gezondheidssituatie te kunnen volgen.

| 43. | | Heeft u op dit moment toegang tot uw medisch dossier via internet?  *(meerdere antwoorden mogelijk)* | |
| --- | --- | --- | --- |
|  |  | | |
| ❒ | | Nee *(ga verder met vraag 46)* | |
| ❒ | | Ja, in het medisch dossier bij de huisarts | |
| ❒ | | Ja, in het medisch dossier van het ziekenhuis | |
| ❒ | | Anders, namelijk |  |

| 44. | | Maakt u gebruik van de mogelijkheid om uw medisch dossier te raadplegen? |
| --- | --- | --- |
|  |  | |
| ❒ | | Nee |
| ❒ | | Ja |

| 45. | | Hoe vindt u het dat u toegang heeft tot uw medisch dossier via internet? |
| --- | --- | --- |
|  |  | |
| ❒ | | Positief |
| ❒ | | Negatief |
| ❒ | | Niet positief, niet negatief |

| 46. | | Ervan uitgaande dat uw medische gegevens goed beveiligd zijn, zou u deze dan via het internet willen kunnen inzien? |
| --- | --- | --- |
|  |  | |
| ❒ | | Ja, ik wil mijn medische gegevens via internet kunnen inzien. |
| ❒ | | Ja, ik wil mijn medische gegevens via internet kunnen inzien en zelf bepalen wie nog meer inzage heeft in mijn gegevens (b.v. welke arts, apotheek of andere zorgverlener). |
| ❒ | | Ja, ik wil mijn medische gegevens via internet kunnen inzien en zelf bepalen wie nog meer inzage heeft in mijn gegevens. Behalve hulpverleners wil ik ook toegang geven aan mijn directe naasten zodat zij beter weten wat er met mij aan de hand is. |
| ❒ | | Nee, voor mij hoeft dat niet. |

| 47. | | Wanneer u via internet de mening van een andere arts (second opinion) zou kunnen vragen, zou u dat dan doen? |
| --- | --- | --- |
|  |  | |
| ❒ | | Nee |
| ❒ | | Ja, maar ik heb dat nog niet gedaan |
| ❒ | | Ja, en ik heb dat ook gedaan |
| ❒ | | Ik weet het niet |

| 48. | | Welke mogelijkheden via internet geven uw arts of ziekenhuis? | | | |
| --- | --- | --- | --- | --- | --- |
| *Kruis één hokje aan op elke regel* | | | **Ja** | **Nee** | |
| 1. Kunnen inzien van eigen medische gegevens | | | ❒ | ❒ | |
| 1. Kunnen opvragen van uitslagen van onderzoeken | | | ❒ | ❒ | |
| 1. Kunnen e-mailen met de behandelend arts(en) | | | ❒ | ❒ | |
| 1. Kunnen e-mailen met de betrokken verpleegkundigen | | | ❒ | ❒ | |
| 1. Kunnen aanvragen en/of verlengen van recepten | | | ❒ | ❒ | |
| 1. Kunnen aanvragen van diagnostisch onderzoek | | | ❒ | ❒ | |
| 1. Kunnen aanvragen van een verwijzing naar een andere specialist | | | ❒ | ❒ | |
| 1. Kunnen maken van een afspraak met de eigen artsen | | | ❒ | ❒ | |
| 1. Kunnen doen van zelfdiagnose-tests | | | ❒ | ❒ | |
| 1. Kunnen plaatsen van een vraag die mijn arts via een forum (anoniem, maar openbaar voor anderen) beantwoordt | | | ❒ | ❒ | |
| 1. Kunnen plaatsen van een vraag die mijn arts beantwoordt en waar andere patiënten op kunnen reageren | | | ❒ | ❒ | |
| 1. Kunnen ‘praten’ (chatten) met lotgenoten die in hetzelfde ziekenhuis behandeld worden voor kanker | | | ❒ | ❒ | |
| 1. Kunnen ontvangen van herinneringen ter ondersteuning van de behandeling | | | ❒ | ❒ | |
| 1. Kunnen melden van klachten | | | ❒ | ❒ | |
| 1. Kunnen aandragen van ideeën voor verbetering van de behandeling | | | ❒ | ❒ | |
| 1. Anders, namelijk: | | |  |  | |
|  |  | | | |  |

| 49. | | | Als uw arts of een gezondheidsorganisatie een (beveiligde) website heeft, waar zou u dan gebruik van maken? | | | | | | | |  |
| --- | --- | --- | --- | --- | --- | --- | --- | --- | --- | --- | --- |
|  |  | | | | | | | | | | |
|  | | | | **Heel graag** | |  |  |  | **Juist niet** | |  |
| *Kruis één van de hokjes aan op elke regel* | | | |  | **1** | **2** | **3** | **4** | **5** |  |  |
| 1. Kunnen inzien van eigen medische gegevens | | | | | ❒ | ❒ | ❒ | ❒ | ❒ |  |  |
| 1. Kunnen opvragen van uitslagen van onderzoeken | | | | | ❒ | ❒ | ❒ | ❒ | ❒ |  |  |
| 1. Kunnen e-mailen met de behandelend specialist | | | | | ❒ | ❒ | ❒ | ❒ | ❒ |  |  |
| 1. Kunnen e-mailen met de betrokken verpleegkundigen | | | | | ❒ | ❒ | ❒ | ❒ | ❒ |  |  |
| 1. Kunnen aanvragen en/of verlengen van recepten | | | | | ❒ | ❒ | ❒ | ❒ | ❒ |  |  |
| 1. Kunnen aanvragen van diagnostisch onderzoek | | | | | ❒ | ❒ | ❒ | ❒ | ❒ |  |  |
| 1. Kunnen aanvragen van een verwijzing naar andere specialist | | | | | ❒ | ❒ | ❒ | ❒ | ❒ |  |  |
| 1. Kunnen maken van een afspraak met de eigen artsen | | | | | ❒ | ❒ | ❒ | ❒ | ❒ |  |  |
| 1. Kunnen doen van zelfdiagnose-tests | | | | | ❒ | ❒ | ❒ | ❒ | ❒ |  |  |
| 1. Kunnen plaatsen van een vraag die mijn arts via een forum (anoniem, maar openbaar voor anderen) beantwoordt | | | | | ❒ | ❒ | ❒ | ❒ | ❒ |  |  |
| 1. Kunnen plaatsen van een vraag die mijn arts beantwoordt en waar andere patiënten op kunnen reageren | | | | | ❒ | ❒ | ❒ | ❒ | ❒ |  |  |
| 1. Kunnen ‘praten’ (chatten) met lotgenoten die hetzelfde ziekenhuis behandeld worden voor kanker | | | | | ❒ | ❒ | ❒ | ❒ | ❒ |  |  |
| 1. Kunnen ontvangen van herinneringen ter ondersteuning van de behandeling | | | | | ❒ | ❒ | ❒ | ❒ | ❒ |  |  |
| 1. Kunnen melden van klachten | | | | | ❒ | ❒ | ❒ | ❒ | ❒ |  |  |
| 1. Kunnen aandragen van ideeën voor verbetering van de behandeling | | | | | ❒ | ❒ | ❒ | ❒ | ❒ |  |  |
| 1. Kunnen bijhouden van zaken waar u last van heeft (zoals vermoeidheid) | | | | | ❒ | ❒ | ❒ | ❒ | ❒ |  |  |
| 1. Kunnen ontvangen van persoonlijk advies afgestemd op uw symptomen | | | | | ❒ | ❒ | ❒ | ❒ | ❒ |  |  |
| 1. Kunnen ontvangen van een overzicht van aanvullende zorgmogelijkheden | | | | | ❒ | ❒ | ❒ | ❒ | ❒ |  |  |
| 1. Kunnen beoordelen van een zorgverlener of zorginstelling | | | | | ❒ | ❒ | ❒ | ❒ | ❒ |  |  |
| 1. Kunnen meedoen aan een online zelfhulpcursus | | | | | ❒ | ❒ | ❒ | ❒ | ❒ |  |  |
| 1. Anders, namelijk: | | | | | ❒ | ❒ | ❒ | ❒ | ❒ |  |  |
|  | |  | | |  |  |  |  |  |  |  |

| **TER AFSLUITING: Samenwerken aan gezondheid** |
| --- |

Hierna staan een aantal uitspraken die iets zeggen over hoe u omgaat met uw ziekte en de behandeling daarvan. Bij elke uitspraak kunt u uw oordeel geven door het nummer te omcirkelen dat het beste bij uw situatie past.

| 50. Wij willen graag iets weten over hoe u met uw gezondheidstoestand omgaat. | |
| --- | --- |
|  |  |

| a. | In het algemeen weet ik van mijn aandoening (en): | | | | | | | | | | | |
| --- | --- | --- | --- | --- | --- | --- | --- | --- | --- | --- | --- | --- |
|  |  | | | | | | | | | | | |
|  | Heel weinig | |  | | | Een beetje | | | Veel | | | |
|  | 0 | 1 | | 2 | 3 | | 4 | 5 | | 6 | 7 | 8 |
|  | ❒ | ❒ | | ❒ | ❒ | | ❒ | ❒ | | ❒ | ❒ | ❒ |

| b. | In het algemeen weet ik over de behandeling en de medicijnen voor mijn aandoening (en): | | | | | | | | | | | |
| --- | --- | --- | --- | --- | --- | --- | --- | --- | --- | --- | --- | --- |
|  |  | | | | | | | | | | | |
|  | Heel weinig | |  | | | Een beetje | | | Veel | | | |
|  | 0 | 1 | | 2 | 3 | | 4 | 5 | | 6 | 7 | 8 |
|  | ❒ | ❒ | | ❒ | ❒ | | ❒ | ❒ | | ❒ | ❒ | ❒ |

| c. | Ik gebruik de medicijnen en voer de behandelingen uit die mijn arts of zorgverlener heeft voorgesteld: | | | | | | | | | | | |
| --- | --- | --- | --- | --- | --- | --- | --- | --- | --- | --- | --- | --- |
|  |  | | | | | | | | | | | |
|  | Nooit | |  | | | Soms | | | Altijd | | | |
|  | 0 | 1 | | 2 | 3 | | 4 | 5 | | 6 | 7 | 8 |
|  | ❒ | ❒ | | ❒ | ❒ | | ❒ | ❒ | | ❒ | ❒ | ❒ |

| d. | Beslissingen over (de behandeling van) mijn aandoening (en) neem ik samen met mijn arts of zorgverlener: | | | | | | | | | | | |
| --- | --- | --- | --- | --- | --- | --- | --- | --- | --- | --- | --- | --- |
|  |  | | | | | | | | | | | |
|  | Nooit | |  | | | Soms | | | Altijd | | | |
|  | 0 | 1 | | 2 | 3 | | 4 | 5 | | 6 | 7 | 8 |
|  | ❒ | ❒ | | ❒ | ❒ | | ❒ | ❒ | | ❒ | ❒ | ❒ |

| e. | Ik ben in staat op met mijn zorgverlener te regelen, dat ik de zorg krijg die ik nodig heb en die past bij mijn cultuur, waarden en opvattingen: | | | | | | | | | | | |
| --- | --- | --- | --- | --- | --- | --- | --- | --- | --- | --- | --- | --- |
|  |  | | | | | | | | | | | |
|  | Nooit | |  | | | Soms | | | Altijd | | | |
|  | 0 | 1 | | 2 | 3 | | 4 | 5 | | 6 | 7 | 8 |
|  | ❒ | ❒ | | ❒ | ❒ | | ❒ | ❒ | | ❒ | ❒ | ❒ |

| f. | Ik regel de (vervolg)afspraken die mijn arts of zorgverlener voorstelt en ga er ook naar toe: | | | | | | | | | | | |
| --- | --- | --- | --- | --- | --- | --- | --- | --- | --- | --- | --- | --- |
|  |  | | | | | | | | | | | |
|  | Nooit | |  | | | Soms | | | Altijd | | | |
|  | 0 | 1 | | 2 | 3 | | 4 | 5 | | 6 | 7 | 8 |
|  | ❒ | ❒ | | ❒ | ❒ | | ❒ | ❒ | | ❒ | ❒ | ❒ |

| g. | Ik houd zelf symptomen en signalen van mijn lichaam in de gaten (zoals bloedsuikerwaarden, gewicht, kortademigheid, pijn, slaapproblemen, stemming): | | | | | | | | | | | |
| --- | --- | --- | --- | --- | --- | --- | --- | --- | --- | --- | --- | --- |
|  |  | | | | | | | | | | | |
|  | Nooit | |  | | | Soms | | | Altijd | | | |
|  | 0 | 1 | | 2 | 3 | | 4 | 5 | | 6 | 7 | 8 |
|  | ❒ | ❒ | | ❒ | ❒ | | ❒ | ❒ | | ❒ | ❒ | ❒ |

| h. | Ik grijp zelf in als mijn lichaam signalen afgeeft en symptomen erger worden: | | | | | | | | | | | |
| --- | --- | --- | --- | --- | --- | --- | --- | --- | --- | --- | --- | --- |
|  |  | | | | | | | | | | | |
|  | Nooit | |  | | | Soms | | | Altijd | | | |
|  | 0 | 1 | | 2 | 3 | | 4 | 5 | | 6 | 7 | 8 |
|  | ❒ | ❒ | | ❒ | ❒ | | ❒ | ❒ | | ❒ | ❒ | ❒ |

| i. | Ik kan omgaan met het effect van mijn aandoening(en) op mijn lichamelijke activiteiten (zoals wandelen, huishoudelijk werk): | | | | | | | | | | | |
| --- | --- | --- | --- | --- | --- | --- | --- | --- | --- | --- | --- | --- |
|  |  | | | | | | | | | | | |
|  | Niet zo goed | |  | | | Redelijk | | | Heel goed | | | |
|  | 0 | 1 | | 2 | 3 | | 4 | 5 | | 6 | 7 | 8 |
|  | ❒ | ❒ | | ❒ | ❒ | | ❒ | ❒ | | ❒ | ❒ | ❒ |

| j. | Ik kan omgaan met het gevolgen van mijn aandoening(en) voor mijn sociale contacten (dat wil zeggen de omgang met andere mensen): | | | | | | | | | | | |
| --- | --- | --- | --- | --- | --- | --- | --- | --- | --- | --- | --- | --- |
|  |  | | | | | | | | | | | |
|  | Niet zo goed | |  | | | Redelijk | | | Heel goed | | | |
|  | 0 | 1 | | 2 | 3 | | 4 | 5 | | 6 | 7 | 8 |
|  | ❒ | ❒ | | ❒ | ❒ | | ❒ | ❒ | | ❒ | ❒ | ❒ |

| k. | In het algemeen lukt het mij om gezond te leven (bijv. niet roken, matig alcoholgebruik, gezond eten, regelmatig bewegen, omgaan met stress): | | | | | | | | | | | |
| --- | --- | --- | --- | --- | --- | --- | --- | --- | --- | --- | --- | --- |
|  |  | | | | | | | | | | | |
|  | Niet zo goed | |  | | | Redelijk | | | Heel goed | | | |
|  | 0 | 1 | | 2 | 3 | | 4 | 5 | | 6 | 7 | 8 |
|  | ❒ | ❒ | | ❒ | ❒ | | ❒ | ❒ | | ❒ | ❒ | ❒ |

| **Misschien willen wij u in de toekomst nogmaals verzoeken om een vragenlijst in te vullen over een ander onderwerp, zodat we kunnen volgen hoe het met u gaat. Deelname is vrijwillig en u kunt stoppen wanneer u wilt, zonder dat dat gevolgen heeft voor uw behandeling of nazorg. Wilt u hieraan meedoen?** | | |  |
| --- | --- | --- | --- |
|  |  | | |
| ❒ | | Ja, ik verleen toestemming aan Profiel om mij uit te nodigen voor deelname aan een volgende vragenlijst. |  |
| ❒ | | Nee |  |

| Als u de volgende vragenlijst per email wilt ontvangen dan kunt u hier uw e-mailadres invullen  (in blokletters) |
| --- |

|  | **@** |  |
| --- | --- | --- |

| Hieronder kunt u alles vermelden wat u nog kwijt wilt, wat u van deze vragenlijst vond en wat eventueel nog over het hoofd is gezien.  Vanwege de anonimiteit kunnen we niet reageren op deze opmerkingen. Mocht u reactie willen, dan kunt u uw opmerking of vraag e-mailen naar [info@profielstudie.nl](mailto:info@profielstudie.nl) |
| --- |

|  |
| --- |

Controleer alstublieft of u geen vragen heeft overgeslagen. Wilt u de vragenlijst alstublieft binnen twee weken. Retourneren in de bijgevoegde antwoordenvelop. Een postzegel is niet nodig!

**Hartelijk dank voor uw medewerking aan dit onderzoek.**

Voor informatie over het onderzoek kunt u ons bereiken per e-mail op [info@profielstudie.nl](mailto:info@profielstudie.nl) of telefonisch:

Mw. Drs. Nicole Horevoorts en mw. Erna de Winter, onderzoeksmedewerkers van Profiel, 088 - 234 68 03.

Mw. Drs. Mies van Eenbergen, onderzoeker van Integraal Kankercentrum Nederland en Tilburg University, maandag t/m donderdag: 06-50 22 03 13.
