# Supplementary material for: Comparing Survivors of Cancer in Population-Based Samples With Those in Online Cancer Communities: Cross-sectional Questionnaire Study
Source: JMIR Cancer. 2022 Mar 8;8(1):e19379. doi: 10.2196/19379 (PMC8941444; doi:10.2196/19379)
Supplement: Multimedia Appendix 2 [file cancer_v8i1e19379_app2.docx]

**Multimedia Appendix 2. Tables with additional data.**

**Table S1.** **Effects of Internet use.**

|  | Population-based (n=233) | OCC  (n=214) | P-value |
| --- | --- | --- | --- |
|  | Yes % | Yes % |  |
| Better informed | 29 | 43 | .002 |
| More visits to doctor | 1 | 1 | 1.00 |
| Discussed information with caregivers | 4 | 10 | .004 |
| Influence on treatment choice | 16 | 21 | .144 |

**Table S2. Results from the multivariate logistic regression on internet search frequency (daily/several times a week vs. several times a month/year or never) during various stages of patients’ journey of illness.**

| **Predictor** | **Wald** | ***P*-value** | **Exp(B) (CI 95%)** |
| --- | --- | --- | --- |
| **Just before diagnosis (n=331)** |  |  |  |
| Type of population | 1.81 | .178 | 1.376 (0.865, 2.189) |
| Age | 1.36 | .243 | 1.252 (0.858, 1.826) |
| Gender | 0.00 | .966 | 1.012 (0.594, 1.722) |
| Education | 0.01 | .916 | 1.018 (0.722, 1.437) |
| Time since diagnosis | 1.22 | .269 | 1.124 (0.914, 1.384) |
| **Right after diagnosis (n=425)** |  |  |  |
| Type of population | 10.30 | .001 | 1.98 (1.306, 3.014) |
| Age | 0.92 | .338 | 1.179 (0.842, 1.649) |
| Gender | 0.52 | .471 | 1.186 (0.745, 1.888) |
| Education | 0.25 | .621 | 0.925 (0.680, 1.259) |
| Time since diagnosis | 2.59 | .107 | 1.175 (0.966, 1.431) |
| **During treatment (n=394)** |  |  |  |
| Type of population | 18.08 | < .001 | 2.535 (1.651, 3.892) |
| Age | 2.68 | .102 | 1.334 (0.945, 1.885) |
| Gender | 0.03 | .858 | 1.045 (0.645, 1.695) |
| Education | 0.13 | .717 | 0.942 (0.681, 1.302) |
| Time since diagnosis | 0.86 | .351 | 1.101 (0.899, 1.347) |
| **During follow-up (n=324)** |  |  |  |
| Type of population | 36.67 | < .001 | 7.891 (4.075, 15.633) |
| Age | 0.08 | .777 | 1.071 (0.667, 1.719) |
| Gender | 1.51 | .219 | 1.506 (0.784, 2.895) |
| Education | 0.70 | .403 | 1.222 (0.764, 1.953 |
| Time since diagnosis | 1.48 | .225 | 1.170 (0.908, 1.508) |

Note. Df = 1.

**Table S3.** **Internet use for online participation in an online cancer community.**

|  | Population-based (n=114) | OCC  (n=214) | P-value |
| --- | --- | --- | --- |
|  | Yes % | Yes % |  |
| Reading (lurking) posts of other patients | 23 | 56 | <.001 |
| Creating a profile | 14 | 74 | <.001 |
| Actively posting text in a blog or discussion group | 5 | 40 | <.001 |

**Table S4. Contact with persons found to be important during cancer journey and media use*.**

|  | Population-based *n*=233 | OCC  *n*=214 | Population-based *n*=233 | OCC  *n*=214 | Population-based *n*=233 | OCC  *n*=214 | Population-based *n*=233 | OCC  *n*=214 | Population-based *n*=233 | OCC  *n*=214 | Population-based *n*=233 | OCC  *n*=214 | Population-based *n*=233 | OCC  *n*=214 |
| --- | --- | --- | --- | --- | --- | --- | --- | --- | --- | --- | --- | --- | --- | --- |
|  | Online contact | | Email | | Facebook | | Twitter | | Whatsapp | | Blog | | Skype | |
|  | Yes (%) | Yes (%) | Yes (%) | Yes (%) | Yes (%) | Yes (%) | Yes (%) | Yes (%) | Yes (%) | Yes (%) | Yes (%) | Yes (%) | Yes (%) | Yes (%) |
| Family | 64 | 69 | 31 | 54 | 7 | 24 | 0 | 1 | 44 | 51 | 1 | 8 | 2 | 5 |
| Friends | 61 | 74 | 28 | 53 | 9 | 29 | 0 | 1 | 45 | 49 | 1 | 9 | 1 | 3 |
| Children | 53 | 47 | 22 | 26 | 3 | 12 | 0 | 1 | 34 | 37 | 1 | 3 | 5 | 5 |
| Colleagues | 31 | 44 | 16 | 35 | 5 | 15 | 0 | 0 | 23 | 29 | 1 | 7 | 1 | 0 |
| Oncology nurse | 31 | 38 | 25 | 30 | 0 | 1 | 0 | 0 | 1 | 2 | 0 | 1 | 0 | 1 |
| Oncologist | 26 | 34 | 14 | 24 | 0 | 1 | 0 | 1 | 1 | 1 | 0 | 0 | 0 | 1 |
| General practitioner | 14 | 22 | 5 | 16 | 0 | 1 | 0 | 0 | 1 | 2 | 0 | 1 | 0 | 1 |
| Other patients | 12 | 51 | 5 | 22 | 4 | 15 | 0 | 0 | 5 | 6 | 2 | 15 | 0 | 1 |
| Members online group | 2 | 32 | 1 | 16 | 1 | 7 | 0 | 0 | 1 | 1 | 1 | 7 | 0 | 1 |

* does not add up to 100%

**Table S5. Treatment and follow up phase that does not apply to respondents from the POP and the OCC group.**

|  | Population-based (n=233) | OCC (n=214) |
| --- | --- | --- |
|  | Not apply  n (%) | Not apply  n (%) |
| Just before the onset of surgery / treatment | 37 (16) | 14 (7) |
| During chemo or hormone therapy | 108 (46) | 58 (27) |
| During radiation therapy | 128 (55) | 92 (43) |
| Follow up | 43 (19) | 79 (37) |

## Table S6 Information searched for on the internet, according to study (%).

| Information searched for | Population-based (n=233) | OCC (n=214) | ranking |
| --- | --- | --- | --- |
|  | % | % |  |
| Type of cancer | 77 | 90 | 1 -1 |
| Consequences of treatment in general | 70 | 86 | 2-3 |
| Treatment | 69 | 88 | 3-2 |
| Cancer and genetic/heritability | 56 | 79 | 4-4 |
| Treatment guidelines | 46 | 63 | 5-6 |
| What I can do myself | 42 | 66 | 6-5 |
| Consequences for sexuality | 41 | 53 | 7-12 |
| Healthcare insurance coverage | 40 | 62 | 8-7 |
| Fatigue | 38 | 61 | 9-8 |
| Financial consequences | 37 | 57 | 10-10 |
| Which hospital is best | 19 | 40 | 11-14 |
| Patient activities in region | 18 | 44 | 12-13 |
| Cancer support groups | 17 | 59 | 13-9 |
| Trials/research | 17 | 54 | 13-11 |
| Alternative medicine | 13 | 33 | 15-16 |
| Where to find a good oncologist | 13 | 33 | 15-16 |
| End of life | 11 | 35 | 17-15 |
| Consequences for future parenthood | 4 | 28 | 18-18 |
